# Supplementary figures and images for: Cortical astrocyte histamine-1-receptors regulate intracellular calcium and extracellular adenosine dynamics across sleep and wake
Source: PLoS Biol. 2025 Oct 2;23(10):e3003376. doi: 10.1371/journal.pbio.3003376 (PMC12490766; doi:10.1371/journal.pbio.3003376)

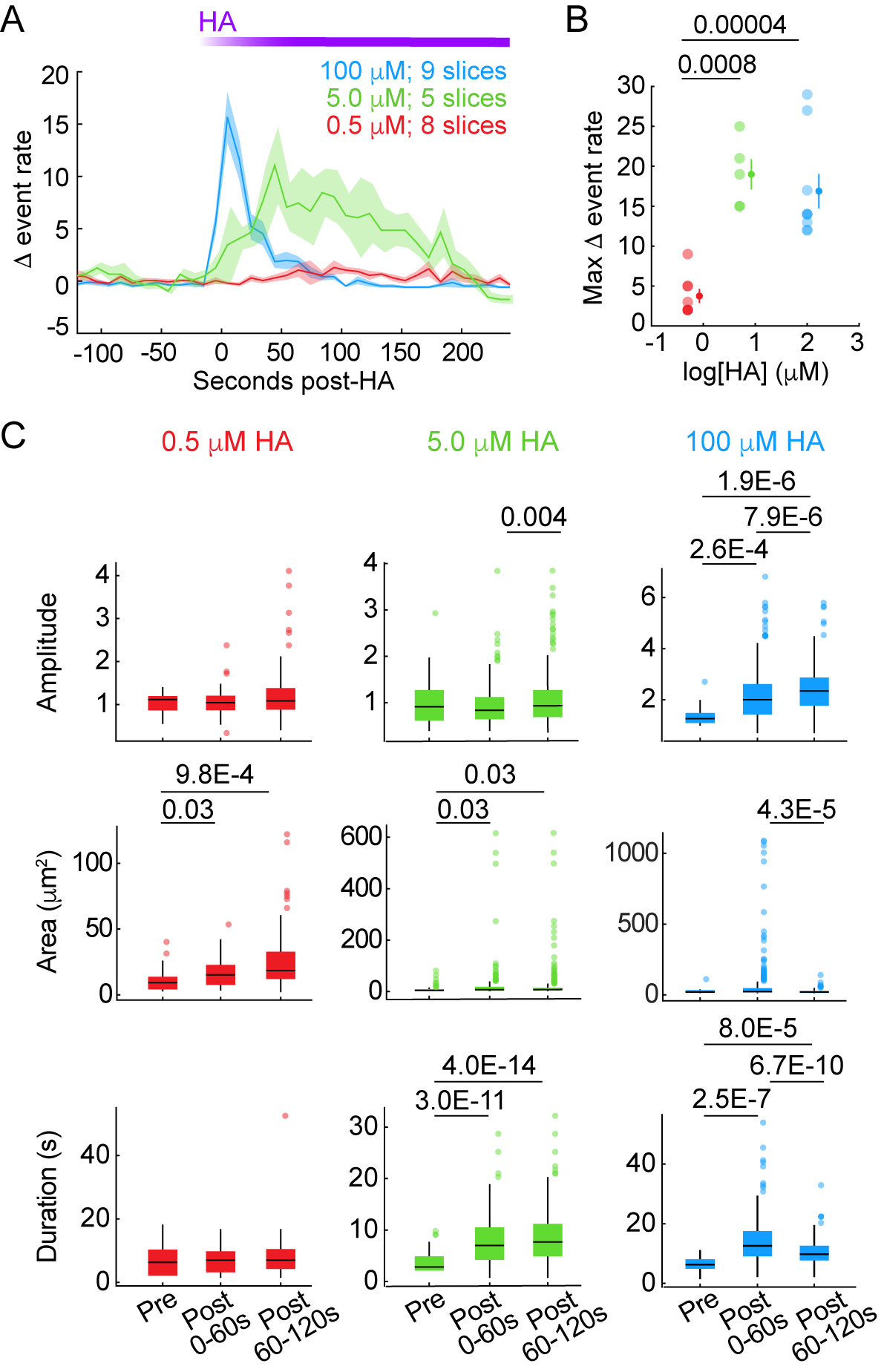

Supplement: S1 Fig — (A) HA-triggered change in Ca2+ event rate (10-s time bins) relative to mean 60 s pre-HA for 0.5, 5, and 100 µM HA, indicated by red, green, and blue traces, respectively. Traces = mean across slices. Shaded error bars = SEM. Purple bar = HA time in recirculating ACSF. (B) Summary of data in A. Maximum change in event rate during 2 min post-HA for each slice, with mean ± SEM at right. p-values via one-sided Wilcoxon rank-sum test. (C) Distribution of event amplitude, area, and duration in 60-s time bins pre- and post-HA addition for 0.5, 5, and 100 µM HA. Each box spans interquartile range (25th–75th percentile), with horizontal line indicating median, whiskers extending to most extreme values within 1.5× the IQR, and outliers plotted individually. p-values via one-sided Wilcoxon rank-sum test. (TIF) [file pbio.3003376.s001.tif]

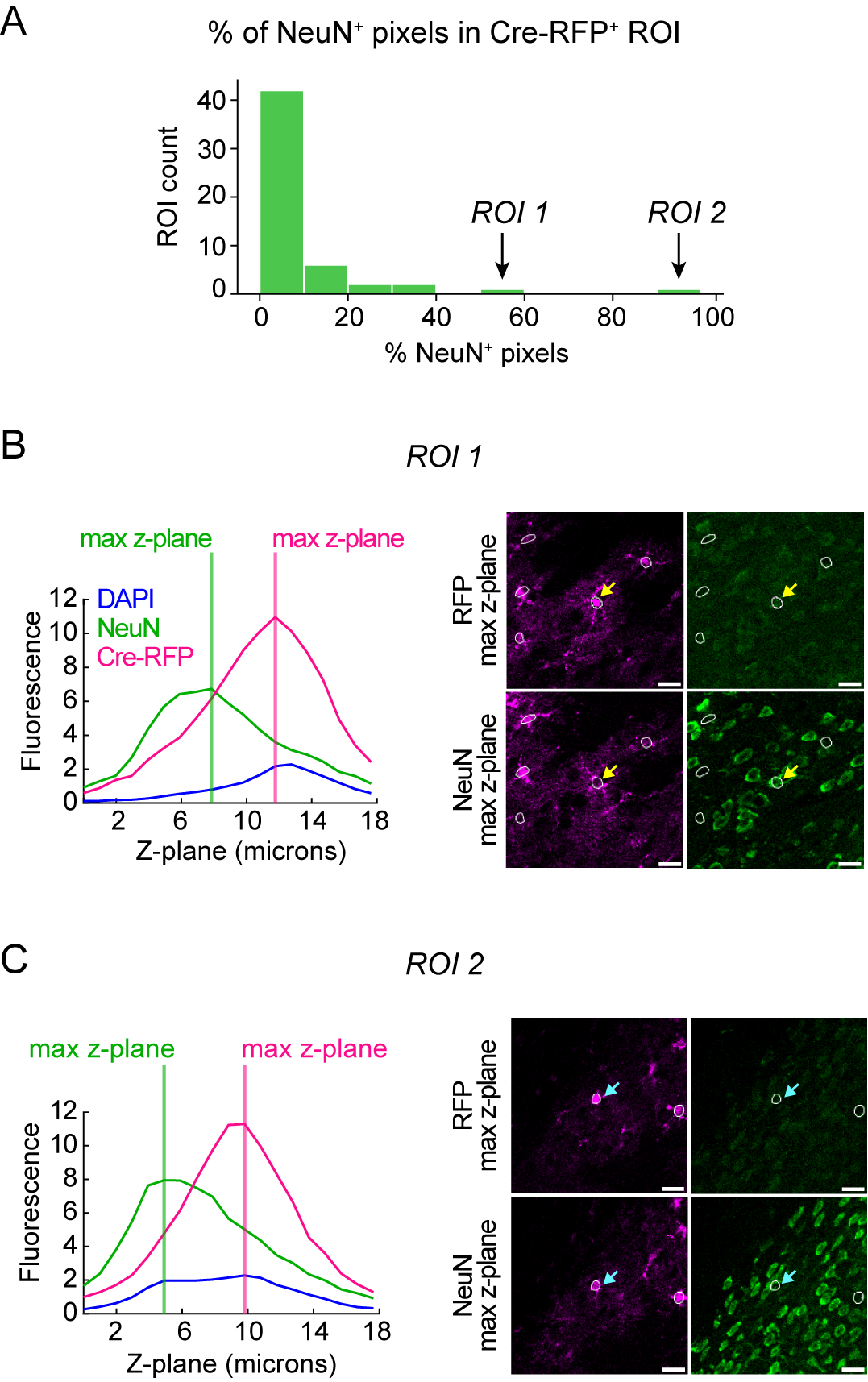

Supplement: S2 Fig — (A) Percentage of Cre-RFP+ astrocyte soma pixels that are NeuN+. Arrows point to two cells with >50% RFP and NeuN overlap, which was quantified by generating summed z-projection of NeuN channel, binarizing the z-projection, and then calculating the % of NeuN+ pixels in somatic ROIs. (B–C) Left: Traces show fluorescence level of DAPI (blue), NeuN (green), RFP (pink) across all z-planes in z-stack for ROI 1 and 2 indicated in panel A. Vertical lines show z-plane of maximum fluorescence for NeuN (green) and RFP (pink), revealing no overlap. Right: Confocal micrographs show RFP and NeuN fluorescence in ROI 1 indicated by yellow arrows and ROI 2 indicated by cyan arrows. Top rows: RFP fluorescence and lack of NeuN fluorescence in z-plane displaying maximum RFP fluorescence. Bottom rows: RFP and NeuN fluorescence in z-plane displaying maximum NeuN fluorescence. (B) ROI 1 displays absence of RFP in NeuN max z-plane. (C) ROI 2 exhibits reduced RFP levels and captures the edges of multiple NeuN+ cells rather than an RFP+ individual neuron. Scale bars = 20 µm. (TIF) [file pbio.3003376.s002.tif]

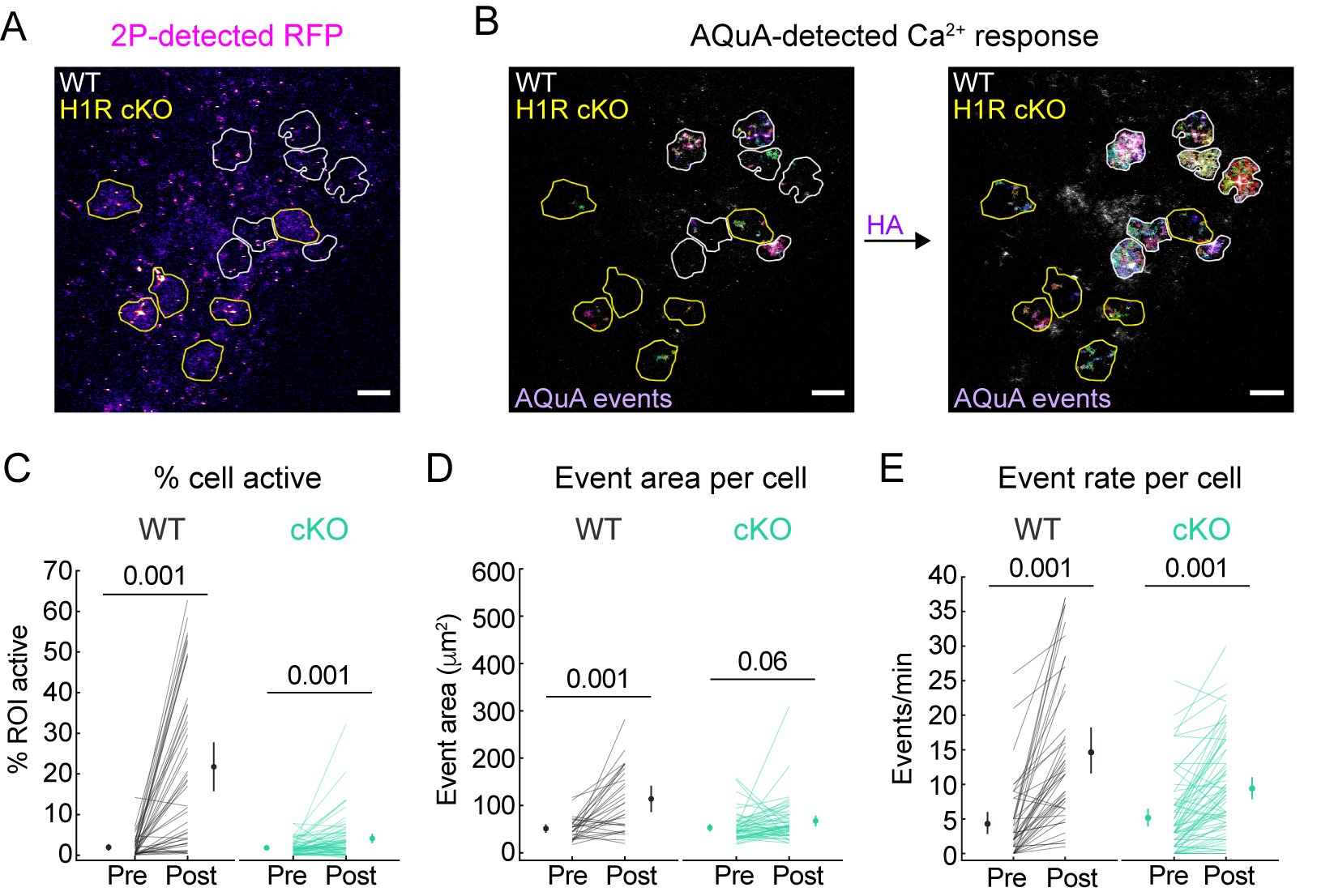

Supplement: S3 Fig — Histamine-1-receptor (H1R) deletion in astrocytes leads to reduced cell area subsumed by histamine (HA)-triggered Ca2+ while leaving wild-type (WT) levels of event synchrony intact. (A) Example z-projection of H1R cKO astrocytes identified via live 2P RFP imaging. H1R cKO astrocytes outlined in yellow. WT astrocytes were identified based on GCaMP6f expression (not shown) and are outlined in white. Scale bar = 50 µm. (B) Sample z-projection of 2P image showing pre- and post-HA AQuA events. WT cells are filled by AQuA events post-HA, while cKO regions of interest (ROIs) exhibit an increased number of discrete AQuA events relative to pre-HA. Pre- and post-HA z-projections include the same number of frames. Scale bars = 50 µm. (C) Mean percent-ROI-active during 1 min pre-HA and 2 min immediately following HA. WT ROIs exhibit increased activity (post – pre = 19.9%). cKO cells show a small increase (post – pre = 2.3%). For C–E, data shown are mean ± 95% CI. (D) Ca2+ event area per cell during 1 min pre-HA and 30 s immediately following HA. WT astrocytes exhibit larger Ca2+ events (post – pre = 59.7 µm2) post-HA, while cKO event area shows no change. Post-HA window is 30 s because area increase is short-lived. (E) Events/min per cell during 1 min pre-HA and 2 min immediately following HA. WT and cKO astrocytes exhibit increased event rate post-HA (WT post – pre = 10.4 events; cKO post – pre = 4.3 events). For C–E, means and 95% CI are estimated via bootstrapping with replacement; p-values are calculated via permutation test on data before bootstrapping. Data in panels C–E collected from 9 slices and 3 mice. (TIF) [file pbio.3003376.s003.tif]

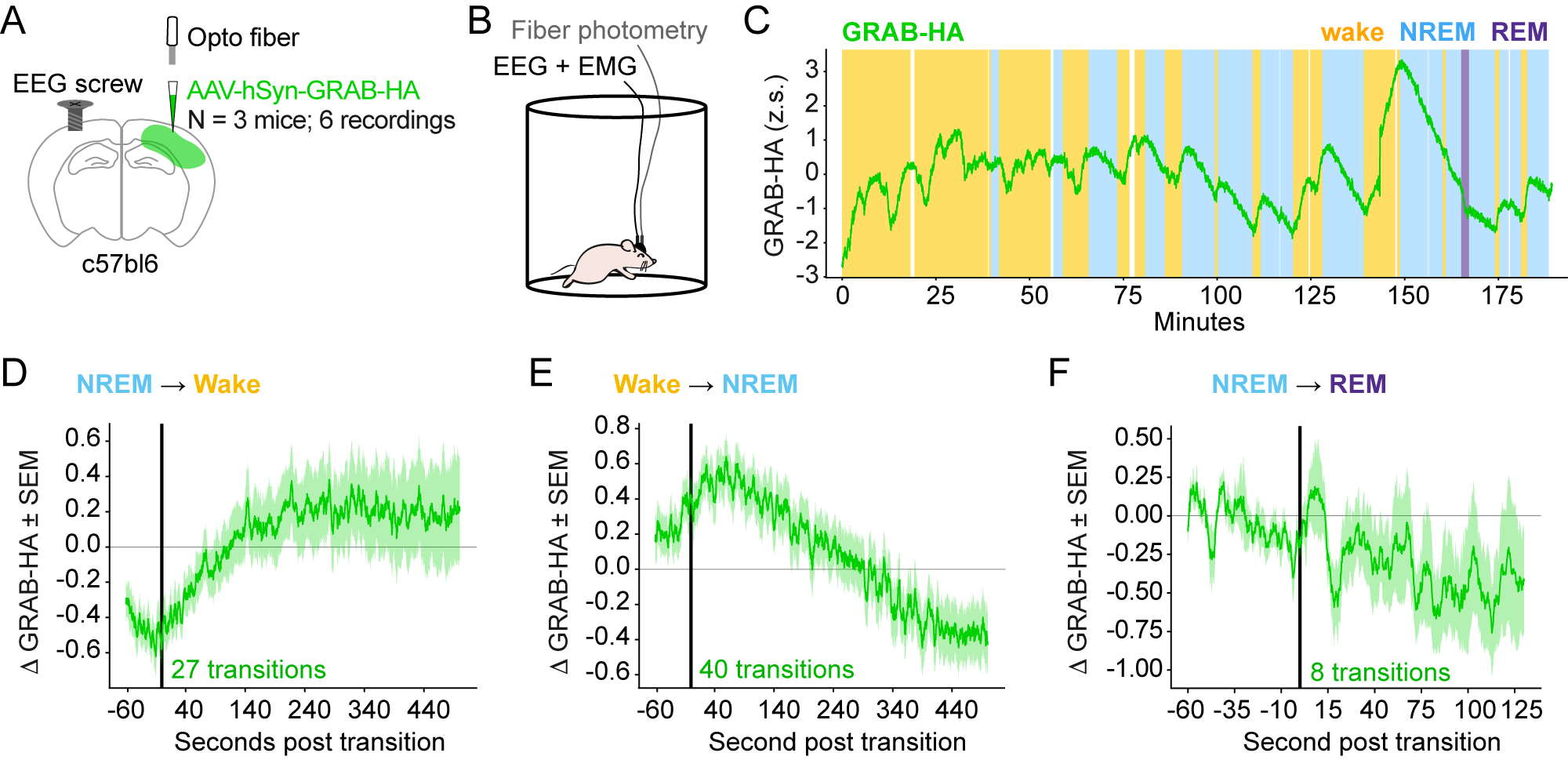

Supplement: S4 Fig — (A) Experimental schematic showing V1 GRAB-HA virus injection with photometry fiber and EEG screw placement in c57bl6 mice. N = 6 recordings across 3 mice. (B) Schematic showing freely moving fiber photometry and electrophysiology recording setup. (C) Example z-scored GRAB-HA photometry trace aligned to color-coded wake (yellow), NREM (blue), and REM (purple) periods. (D–F) Event-triggered averages of change in GRAB-HA fluorescence relative to mean of pre-transition period. Trace = mean GRAB-HA activity. Shaded error bar = SEM. (D) NREM-to-wake transitions spanning 560 s. Trace shows mean of 27 transitions. (E) Wake-to-NREM transitions spanning 560 s. Trace shows mean of 40 transitions (F) NREM-to-REM transitions spanning 180 s. Trace shows mean of 8 transitions. Note: too few REM-to-wake transitions (3 total) identified for quantification of mean GRAB-HA levels. (TIF) [file pbio.3003376.s004.tif]

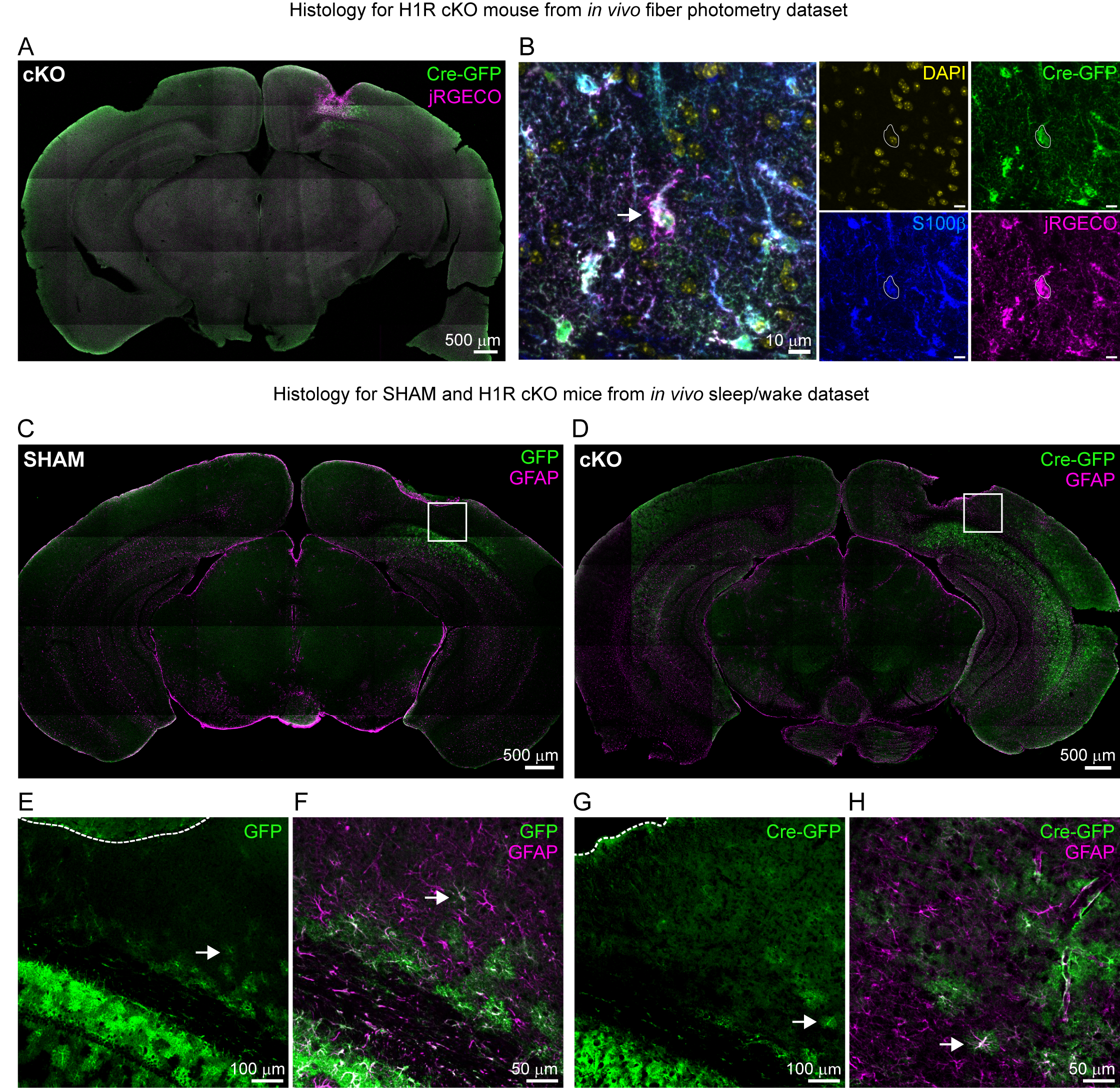

Supplement: S5 Fig — (A) Representative confocal micrograph of histamine-1-receptor (H1R) cKO brain slice for fiber photometry datasets. Astrocytic Cre-GFP (green) and jRGECO (magenta) is expressed below photometry fiber track in right hemisphere. EEG screw track visible in contralateral hemisphere. Scale bar = 500 µm. (B) Left: confocal micrograph showing DAPI (yellow), Cre-GFP (green), jRGECO (magenta), and S100β (blue) expression below photometry fiber track shown in A. White arrow indicates GFP+/jRGECO+/S100β+ astrocyte. Scale bar = 100 µm. Right: separated confocal channels showing DAPI, GFP, jRGECO, and S100β expression in outlined astrocyte indicated with white arrow in left image. Scale bars = 10 µm. (C–D) Representative confocal micrographs of sham (C) and cKO (D) brain slices for dataset shown in Fig 8. Astrocytic Cre-GFP or GFP (green) and GFAP (magenta) expression below EEG screw track in right hemispheres. White boxes outline areas shown in E–H. Scale bars = 500 µm. (E–H) Confocal micrographs in E, F and G, H show GFP+ (green) and GFAP (magenta) expression in outlined regions in C and D, respectively. (E, G) Confocal micrograph showing astrocytic Cre-GFP expression in cortex and hippocampus below EEG screw track. White arrows indicate GFP+ astrocyte shown at a higher magnification in F and H. White dotted line = tissue edge of EEG screw track. Scale bars = 100 µm. (F, H) Higher magnification confocal micrograph showing GFP+ (green) and GFAP (magenta) co-expression in cortical astrocytes below EEG screw track. White arrows in F and H indicate GFP+/GFAP+ astrocyte in cortex shown by white arrows in E and G, respectively. Scale bars = 50 µm. (TIF) [file pbio.3003376.s005.tif]

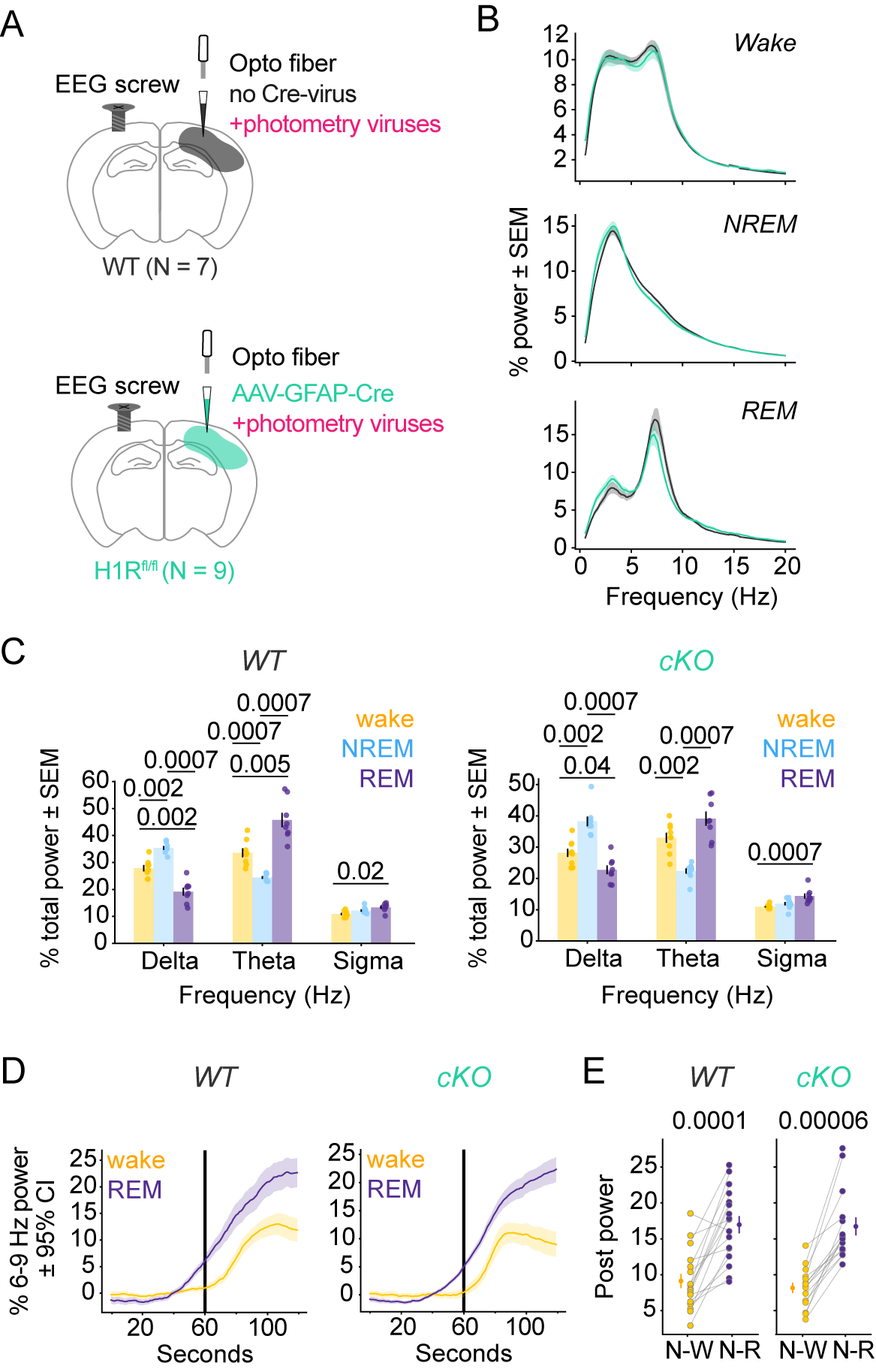

Supplement: S6 Fig — (A) Mouse surgery schematics show location of EEG screw, photometry fiber, and virus injections in WT and H1R cKO mice. Photometry fiber was implanted over V1 injection site for astrocytic jRGECO virus ± extracellular GRAB-Ado and astrocytic Cre or sham virus. EEG screw was implanted in contralateral V1. WT cohort included c57bl6 and H1Rfl/fl mice. Color code (cKO in cyan, WT in gray) used in panels B–E. (B) Power spectral density (PSD) plots show WT (gray) and cKO (cyan) % power relative to total power across 1–20 Hz during wake, NREM, and REM. PSDs calculated via multitaper spectrogram. Traces = mean across mice; shaded error bar = SEM. (C) WT (left) and cKO (right) relative band power (% of total power in each state) for delta (1–4 Hz), theta (6–10 Hz), and sigma (10–15 Hz) during wake (yellow), NREM (blue), and REM (purple). Bars show overall mean and overlaid data points show mean per mouse. Error bars = SEM. p-values via Wilcoxon rank-sum test with Benjamini–Hochberg correction for multiple comparisons. (D) Event-triggered averages show change relative to mean of pre-transition period in theta power around NREM-wake (yellow) and NREM-REM (purple) transitions spanning 120 s for WT (left) and cKO (right). Traces = mean across mice; shaded error bars = 95% CI. (E) Summary statistics for data in D show increased theta power at REM-onset relative to wake-onset for WT (left) and cKO (right). Scattered data points show mean (per recording) % theta power during 60 s post transition for NREM-to-wake (yellow) and NREM-to-REM (purple). Data points with error bars = overall mean ± SEM. p-values via one-sided Wilcoxon rank-sum test. (TIF) [file pbio.3003376.s006.tif]

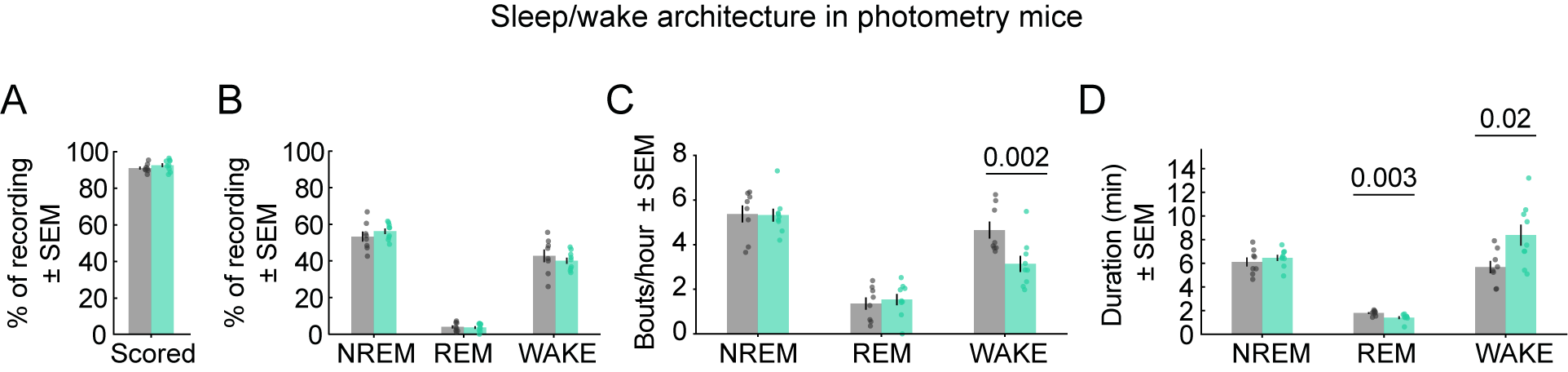

Supplement: S7 Fig — (A) % of individual recordings scored as wake, NREM, or REM. For A–D, bars show overall mean and overlaid data points show mean per mouse ± SEM (WT: 90.7 ± 0.8%, cKOs: 92.2 ± 1.0%). p-value calculated via one-sided Wilcoxon rank-sum test. (B) Mean % time (relative to total time scored) in wake, NREM, and REM. % time per state for WT: 42.8 ± 3.4 wake, 53.3 ± 2.7 NREM, 4.0 ± 0.8 REM; cKO: 40.1 ± 1.7 wake, 56.2 ± 1.5 NREM, 3.6 ± 0.7 REM. One-sided Wilcoxon rank-sum test detects no difference in % time in sleep/wake. (C) Mean bouts per hour for wake, NREM, and REM. Bouts per hour for WT: 4.7 ± 0.4 wake, 5.4 ± 0.4 NREM, 1.4 ± 0.3 REM; cKO: 3.1 ± 0.4 wake, 5.3 ± 0.3 NREM, 1.5 ± 0.3 REM. p-value via one-sided Wilcoxon rank-sum test. (D) Mean bout duration in minutes for wake, NREM, and REM. Mean bout duration (min) for WT: 5.7 ± 0.5 wake, 6.1 ± 0.4 NREM, 1.8 ± 0.1 REM; cKO: 8.38 ± 0.9 wake, 6.5 ± 0.3 NREM, 1.4 ± 0.1 REM. (TIF) [file pbio.3003376.s007.tif]
