# Supplementary material for: Cortical astrocyte histamine-1-receptors regulate intracellular calcium and extracellular adenosine dynamics across sleep and wake
Source: PLoS Biol. 2025 Oct 2;23(10):e3003376. doi: 10.1371/journal.pbio.3003376 (PMC12490766; doi:10.1371/journal.pbio.3003376)
Supplement: Attachment — Submitted filename: Response to Reviewers.pdf [file pbio.3003376.s014.pdf]

Response to Reviewers

We thank the reviewers for their thoughtful reading and thorough feedback. We believe that addressing reviewer comments has significantly bolstered the quality of the manuscript. We present a point-by-point response to the reviewers' comments below (starting in blue text), but start with a section that highlights major comments from all reviewers.

All three reviewers emphasized the limitations of using an automated classifier (Hidden-Markov model) to code sleep/wake states, particularly REM sleep, since we omitted EMG analysis from REM classification in a subset of recordings and since many of our key *in vivo* results are REM-specific. After reanalysis, we agree that our automated REM classification method was not ideal and prone to error. We have made two changes to thoroughly address this issue.

First, we excluded recordings (10 WT, 8 cKO) if the EMG signal did not exhibit clear gamma (65–100 Hz) dynamics with a reasonable signal-to-noise ratio and a relatively stable baseline. Second, we visually inspected all sleep/wake coding results produced by the HMM automated classifier and manually edited REM classification based on thresholds defined in consultation with members of the sleep-focused Ganguly lab, here at UCSF. We largely limited our manual edits to REM classification and left automated NREM and wake classification mostly intact because, while automated classifiers can make errors, manual classification has its own limitations including experimenter biases and subjective drift.

Response Figure 1

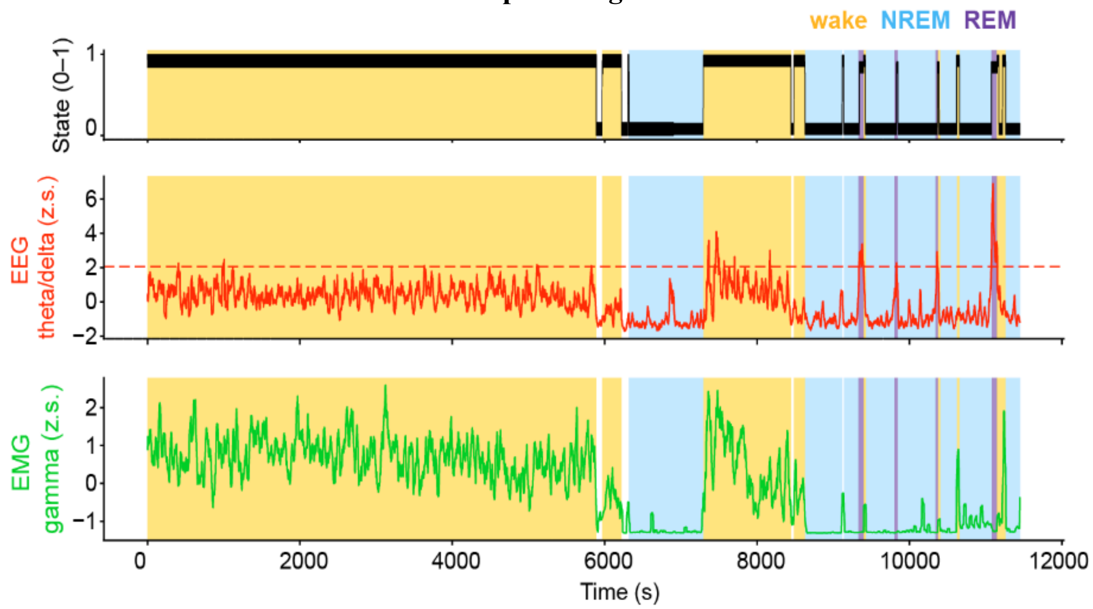

The new methodological details for manual sleep/wake coding are as follows, and are detailed in the paper. EMG gamma (65–100 Hz), EEG theta (6–9 Hz) and EEG delta (0.5-4 Hz) were calculated via multitaper spectral analysis using a sliding 30s window with a 1s step and 29 tapers. Then, sleep/wake scoring results from submission 1 (black trace in Response Figure 1) were plotted alongside z-scored EEG theta/delta ratio (red trace in Response Figure 1) and z-scored EMG gamma power (green trace in Response Figure 1). We visually identified peaks in theta/delta that coincided with low EMG gamma to manually denote REM bout start and end times. See sample recording (Response Figure 1) with manually edited sleep/wake bouts overlaid on z-scored EMG gamma power and z-scored theta/delta trace with dotted red line showing threshold for REM classification. Manually identified REM periods (purple blocks in Response Figure 1) had the following criteria:

1. followed NREM period
2. exhibited a theta/delta ratio > median + 2 standard deviations
3. exhibited little to no EMG gamma power.

Additionally, based on Reviewer 2's emphasis on the lack REM-to-wake transitions in our data, we paid careful attention to increases in EMG gamma power that closely followed REM-coded theta/delta peaks, and classified these periods as wake. Using this method, we identified many more brief awakenings that followed REM, consistent with known mouse sleep/wake behavior[1–3]. That said, we still identified REM-to-NREM transitions (see Response Figure 2: top row, second panel), which is inconsistent with known mouse sleep/wake dynamics, as mentioned by Reviewer 2. In the Methods, we now state that we believe this is "*likely due to the seconds-level temporal resolution of our multitaper analysis, which could smooth out brief elevations in EMG gamma, leading to detection of fewer brief awakenings. By using a 30s window with a 1s step we decided to sacrifice temporal resolution for frequency resolution for accurate HMM detection of state transitions that span tens of seconds to minutes*", which should be sufficient for analyzing jRGECO and GRAB-Ado dynamics that fluctuate on a seconds-long timescale. Since EMG gamma was calculated the same way for all recordings, this caveat should affect WT and cKO datasets equally. However, because of this caveat, we do not further analyze REM-to-NREM transitions, since these periods might be contaminated with microarousals that we were unable to detect. Similarly, we do not further analyze microarousals.

After excluding experiments without interpretable EMG recordings and manually refining REM and wake classifications, we observed marked improvements in sleep/wake scoring accuracy. This is demonstrated by comparing wild-type EMG gamma dynamics before (submission 1) and after (resubmission) rescoring—see plots in Response Figure 2. Note that this direct submission 1 vs resubmission comparison is limited to one *in vivo* dataset: the WT data shown in Figure 8 of the manuscript.

Response Figure 2

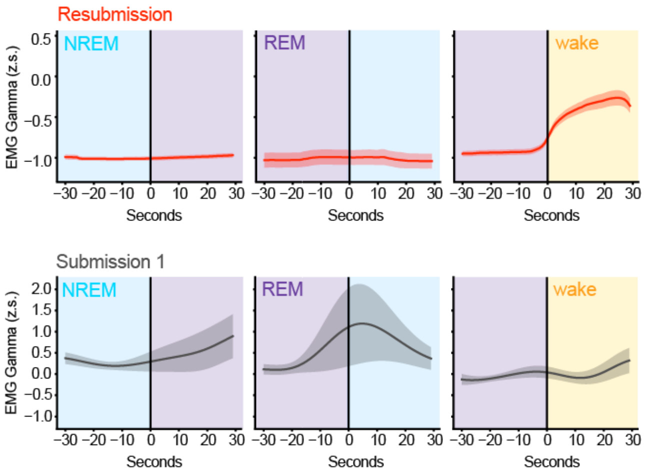

Overall, manual edits reveal REM dynamics that are more consistent with published REM hallmarks in mice. In resubmission data, EMG gamma is low during REM and increases specifically at wake-onset. In contrast, submission 1 plots show an increase in gamma power at NREM-to-REM transitions and just prior to the transition from REM to NREM, which likely reflects contamination of submission 1 REM data with wake bouts and unidentified REM-to-wake transitions. We identified many more REM-to-wake transitions by visual inspection of EMG gamma activity—sufficient to quantify a clear increase in EMG gamma power at this transition (Response Figure 2: top row, third panel). Finally, REM-specific power spectrum density (PSD) plots from reanalyzed datasets exhibit more power concentrated around the theta band, which is consistent with published REM PSDs from mice. This is clear in the comparison of WT data from submission 1 and resubmission (Response Figure 3).

Response Figure 3

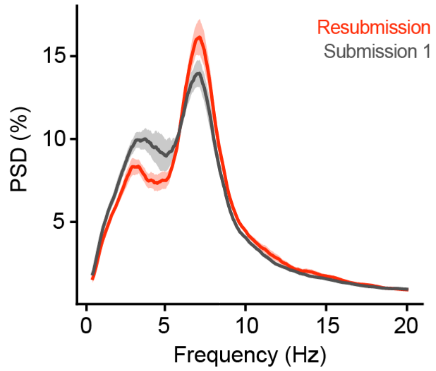

The overarching story of our manuscript has changed after manually editing REM and wake classification. As a result, we have edited existing figures, added new main and supplemental figures, and made substantial edits to the text throughout the manuscript. Overall, we believe the changes in sleep/wake coding have significantly bolstered our *in vivo* findings, and we express our sincere appreciation for the reviewers' suggestions that led to our careful reanalysis.

Reviewer 1

Taylor et al. present an interesting, well-conducted and well-presented study. They demonstrate direct effects of histamine on astrocytic calcium signaling through H1R and further show that astrocytic H1R deletion causes changes to astrocyte calcium responses in REM sleep as well as influencing adenosine levels in sleep.

We appreciate Reviewer 1's positive summary of our manuscript. We address more specific comments below.

Comments/suggestions:

(p 9, results). The authors show by measuring sleep with an EEG electrode in V1 in an area with localized knockdown of H1R that the knockout shifts the measured sleep/wake ratio. However, when measuring in the contralateral cortex to the viral injection no difference in sleep/wake ratio is found. In the discussion they propose that this observation may be a representation of local sleep. This is really an intriguing finding. However, the sleep/wake classification is based on EEG/EMG, and EMG in some cases are potentially missing (according to the methods section - not sure whether this comment relates to these observations). When examining Fig 6 spectrograms one gets the impression that there are subtle differences in particular in REM sleep, even though the aggregated statistics show no differences.

Here, Reviewer 1 questions S/W differences measured in cortices contralateral versus ipsilateral to cKO astrocytes, due lack of EMG inclusion in some sleep/wake scoring. After visually inspecting sleep/wake scoring and manually editing REM classification based on EMG gamma power (as described in detail above), we still find different sleep/wake phenotypes depending on whether cKO astrocytes are contra- or ipsilateral to the EEG screw used to track sleep/wake. However, the differences are much more subtle after reanalysis (compare Figure 8 and S7). Considering experimental conditions were not set up to test whether cKO astrocytes can drive different sleep/wake dynamics in ipsi- versus contralateral hemispheres, we now suggest in the text that sleep/wake differences between these cohorts are likely due to two distinct experimental setups, including viruses expressed and an additional fiber photometry cable tethered to the head mount of individual mice.

When referring to spectrograms, we assume Reviewer 1 means the power spectral density plots in panel Figure 8E (previously 6D), since we do not show any spectrograms in this figure. The subtle differences in REM spectral density plots do not persist after manually editing REM classification and reanalyzing sleep/wake data. As in submission 1, we do not observe any significant differences between WT and cKO state-specific spectral properties, suggesting that astrocytic H1R does not modulate spectral properties of sleep/wake states when measured via an EEG screw in the ipsilateral hemisphere relative to Cre-virus transduction.

1) With the automated classifier, with potential small differences in REM spectral components and lack of EMG in some recordings, how sure can the authors be that these differences in sleep/wake are not due to a mislabeling of for instance wake vs REM by their automated classifier? Were EMG recordings present in all the recordings in Fig 6 and Supplementary figure 5? This aspect could maybe be discussed, alternatively, could other thresholds or algorithms for sleep scoring be used to verify the findings?

Based on multiple reviewer comments along these lines, we visually inspected sleep/wake coding generated by the automated classifier and manually edited REM and wake classification. Please see introductory section of this document for more details. In our resubmission, EMG recordings were used in sleep/wake scoring for <u>all</u> recordings (including data in Figures 8, S6, and S7 which were previously shown in 6 and S5).

2) Were EEG recordings on ipsi- and contralateral side to the viral induced knockout of H1R recorded simultaneously in the same mouse, or in separate experiments?

Ipsi- and contralateral EEG recordings were recorded in separate experiments. In the Results section of our resubmitted manuscript, we have added the following to clarify experimental setups:

"*In a separate experiment from the photometry experiments above, we targeted genetic deletion of astrocytic H1R to the cortex and examined cortical arousal and sleep/wake behavior.*"

Sleep, and in particular REM sleep, is characterized by large hemodynamic changes that influence fluorescence recordings with relatively low SNR fluorescent sensors.
3) If I understand the methods section correctly, all recordings of the GRAB-Ado have been corrected for with the "isosbestic signal" (405nm stimulation)? I'm not intimately familiar with these systems, but I presume there is a sequential activation of the LEDs of different colors? Some more details on this should be provided in methods.

Yes, the isosbestic signal (405 nm channel) was used to normalized both GRAB and jRGECO signals. We have clarified photometry signal acquisition in the Methods section:

"*Fiber photometry was performed on a Tucker-Davis Technologies RZ10X processor (TDT) outfitted with the Lux photometry module (integrated 405, 465, and 560 nm LEDs; photosensors; low-autofluorescence 400 µm, 0.57 NA patch cords; Doric Lenses). All three LEDs were driven concurrently but each was sinusoidally modulated at a unique carrier frequency (lock-in multiplexing), allowing deconvolution of the 405 nm "control," 465 nm (jRGECO), and 560 nm (GRAB-Ado) channels at the photoreceiver. Demodulated emission signals were digitized at 1017 Hz and low-pass filtered at 6 Hz in TDT Synapse software and collected through bandpass filters: 500–540 nm for 405/465 nm excitation, and 580–680 nm for 560 nm excitation.*"

After acquisition, standard signal processing steps[4] were applied to normalize jRGECO and GRAB signals:

1. Low-pass filter (< 3 Hz, Kaiser window FIR)
2. Smooth signals (Savitzky-Golay, 5 s window)
3. Detrend signals (linear regression)
4. Fit 405 nm signal to 465 and 560 nm signals (least squares)
5. Subtract fitted 405 signal from 465 and 560 signals
6. Z-score normalized 465 and 560 signals

Photometry traces before and after signal processing steps are illustrated on the following page. For the sake of brevity, and since similar methods are used in other publications[5–7], we have not included this figure in the manuscript.

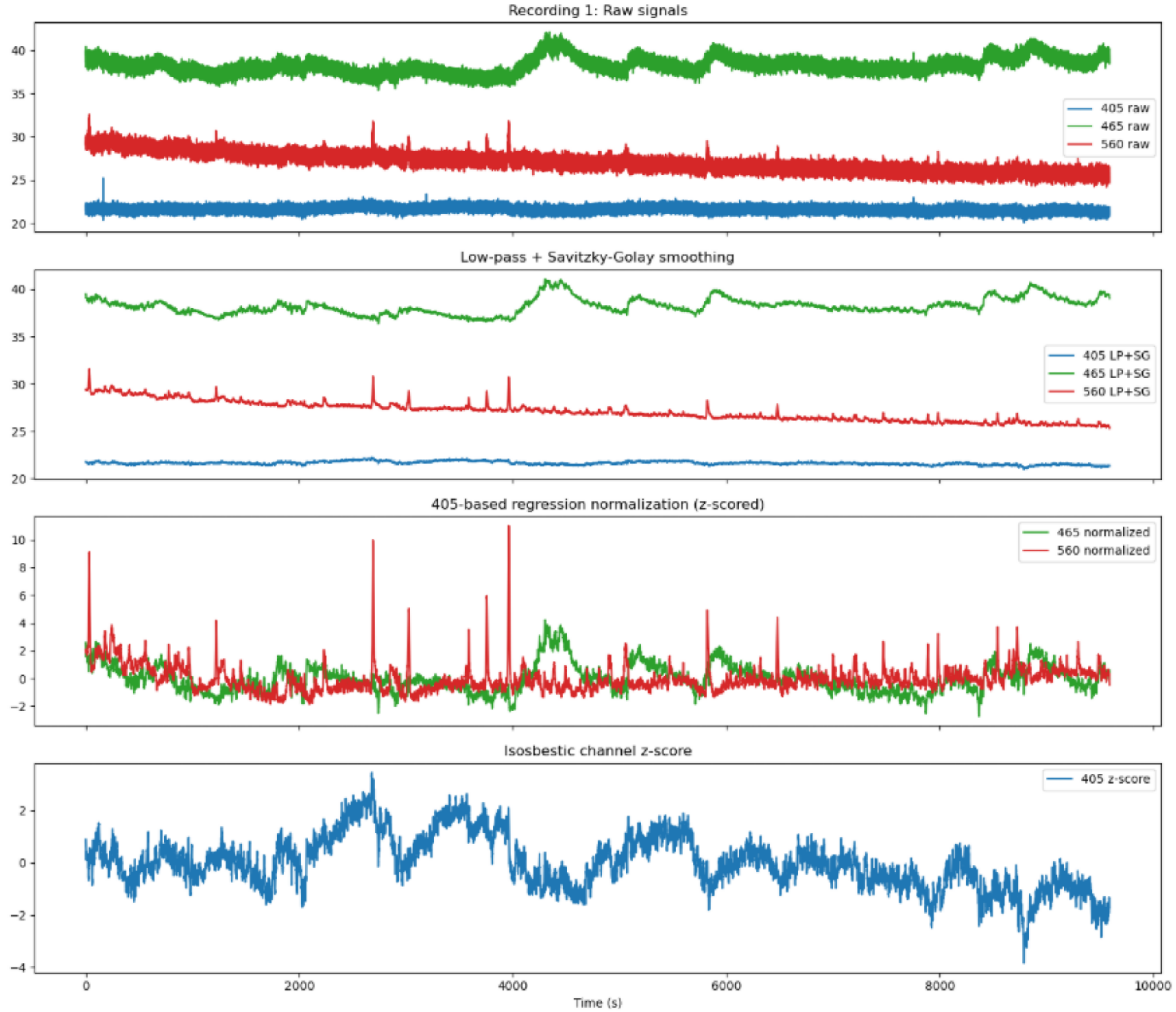

Recording 1: Raw signals
405 raw
465 raw
560 raw
Low-pass + Savitzky-Golay smoothing
405 LP+SG
465 LP+SG
560 LP+SG
405-based regression normalization (z-scored)
465 normalized
560 normalized
Isosbestic channel z-score
405 z-score
Time (s)

4) The absorption spectrum of hemoglobin varies quite significantly over the range light used (405/465/560). Can we be sure that this method adequately removes hemodynamic artifacts from the recordings? Or could even the correction introduce artifacts? Moreover, in a hypothetical scenario where the H1R knockout changes vascular dynamics in REM sleep, could this affect the results?

We appreciate Reviewer 1's important point regarding hemodynamic interference in our photometry data, which we did not fully address in our initial submission. The absorption spectrum of hemoglobin overlaps with 405 nm and the excitation and emission spectrum of the indicators used in our experiments. As a result, increased blood volume, which leads to greater hemoglobin absorption, reduces the amount of light reaching the photosensors[8]. This causes signal decreases in the 405 and 465 channels. The 560 channel is more complex—because deoxygenated hemoglobin absorbs more at 560 nm than oxygenated hemoglobin, increased blood flow and oxygenation may result in slightly less signal attenuation at the photosensors.

Given that hemodynamics and other factors (e.g., motion, photobleaching, autofluorescence) can differentially affect jRGECO and GRAB signals, no single normalization approach can fully correct both signals without introducing artifacts. To remain consistent with the literature, we have retained our original signal normalization method in both submissions. This normalization method, which fits and subtracts the 405 signal from the 465 and 560 signals, accounts for hemodynamics since the 405 nm channel tracks changes in blood flow due to the hemoglobin absorption peak around this wavelength. Nevertheless, we now explicitly discuss in the text how dynamic changes in blood flow during sleep/wake cycles could influence our data, and how astrocytic H1R deletion may alter hemodynamics, which could drive the genotype-specific jRGECO and GRAB-Ado effects that we observe. We have added the following text to the Discussion:

"*…it is possible that brain hemodynamics contribute to the increase in astrocytic jRGECO fluorescence that we observed at REM-onset. Considering oxygenated hemoglobin absorbs less 560 nm light than 405 nm (wavelength used to normalize photometry signals), it is possible that increased oxygenation at REM-onset leads to over-correction of the 560 nm signal, since negative deflections occurring at REM-onset in the 405 nm signal are subtracted from both 560 and 465 channels (see Methods).*

Nevertheless, our results call into question the assumption that sleep- and wake-specific astrocyte Ca^{2+} *dynamics are homogeneous across the cortex…*"

"*…because REM transitions are accompanied by large shifts in blood flow—and both histamine and astrocytes influence cerebral hemodynamics—genotypic differences in vascular dynamics may partially contribute to the REM-specific fluctuations in GRAB-Ado fluorescence that we observe. For example, H1R deletion could reduce blood flow dynamics around REM transitions, which could explain the larger changes in GRAB-Ado fluorescence in the cKO. This effect could arise from signal normalization via subtraction of the 405 nm signal, which is sensitive to hemodynamic changes due to strong hemoglobin absorption at this wavelength.*"

5) Along the same lines, the authors report a surprisingly big increase in calcium signaling in REM sleep compared to NREM sleep - different from previous reports from other parts of the cortex. Could the astrocytic calcium signal be affected by hemodynamic artifacts, OR the correction performed using the 405 nm signal in such a way that it exaggerates the level of calcium signaling in REM sleep? If the 405/"isosbestic signal" is more affected by hemoglobin absorption than the 465 nm and 560 nm line, would not the compensation artificially elevate both the 465 and 560 line?

After manually editing REM classification, we still see calcium increase at the transition from NREM to REM (Figure 6H), although this increase is much smaller than that observed in our initial submission (likely due to wake contamination of REM bouts in submission 1 analysis).

Considering blood flow and oxygenation both increase at REM-onset[9,10], it is possible that hemodynamics might introduce artifacts in the data, especially since the 405, 465 and 560 channels are differentially affected by hemodynamics (see response above). We now discuss the possibility that hemodynamics might introduce artifacts in our jRGECO photometry data (see response above), potentially leading to discrepancies between our data and two-photon based studies that show decreased calcium during REM in other somatosensory cortices.

That said, a neurobiological—rather than technical—explanation for the discrepancy between our work and published astrocyte studies in other cortices is as follows: In contrast to neuronal activity in non-visual cortical areas, V1 neuronal activity increases during REM[3], which could drive increased surrounding astrocyte calcium activity, since glutamate can drive V1 astrocyte calcium[11].

6) To the best of my knowledge, there are no other good methods to correct for hemodynamics artifacts than what the authors have done. However, a (supplementary) figure showing all the different signals (while stimulating with different LEDs) including transitions to and from REM, and the steps of signal processing performed would be a significant improvement. Moreover, to discuss this technical aspect in the discussion would improve the manuscript, and also raise the awareness of hemodynamic artifacts in sleep recordings (seems like many publications do not at all address this issue).

While we appreciate Reviewer 1's real concerns about how photometry signals are normalized and interpreted—particularly across state transitions—we have decided against including a supplementary figure showing signal processing steps for the sake of brevity. We do recognize the importance of full transparency in signal processing and normalization, and thus have added more detailed descriptions of signal acquisition and analysis in the Methods section. We also now explicitly discuss potential hemodynamic influences in our data, which we agree are often insufficiently addressed in photometry-focused publications.

Reviewer 2

In this manuscript by Taylor et al., the authors sought to determine if the wake-promoting neuromodulator histamine can act through astrocytes in the primary visual (V1) cortex to impact cortical arousal and sleep/wake behavior. These studies address important, fundamental questions regard the role of glia in sleep and wakefulness. Using a combination of ex vivo and in vivo preparations paired with dynamic imaging, electrophysiological, and genetic techniques, they report histamine differentially alters astrocyte intracellular calcium via the histamine H1 receptor (H1R) across sleep/wake states as well as adenosine activity specifically around rapid eye movement (REM) sleep. They also reported that conditional knockout (cKO) of astrocytic H1R in V1 had minimal effects on sleep/wake behavior but caused a small increase in time spent in wakefulness contrary to what would be predicted from this manipulation. The studies were generally well-designed, imaging aspects of these experiments were rigorous and technically well-executed, and the manuscript is clearly written with sound rationale. However, my primary concerns center around aspects of the execution of sleep/wake analyses and experimental design which could lead to inaccurate interpretations of the data. I detail these and other concerns below:

We thank Reviewer 2 for their positive summary and thorough evaluation of our manuscript. We believe that our manuscript has been significantly improved by addressing the valid concerns outlined below.

ESSENTIAL

1) The authors use a combination of multitaper spectral analysis and a Hidden Markov Model to automatically assign sleep/wake states to electroencephalographic (EEG) activity (i.e., score). It does not

seem the electromyography (EMG) was used to support state detection except for in some REM sleep bouts. One major issue is that it does not appear that this autoscoring was manually/visually verified and corrected by an experimenter. Autoscoring approaches are notoriously known for inaccurately scoring REM sleep (i.e., distinguishing REM sleep from wakefulness) as well as state transitions even with additional measures like EMG. Given the data presented, there are a few indications that there are errors in state designation primarily with respect to REM sleep:

Please see above for more methodological details and representative figures demonstrating changes to our results after manual edits to sleep/wake scoring.

a. First, mice typically transition out of REM sleep directly to wakefulness (even if brief) before entering NREM sleep. Direct REM-to-NREM sleep transitions are not typically observed. However, the hypnograms shown in Figures 5B and 7B show few (if any) denoted REM episodes immediately followed by wakefulness. The authors also state that too few REM-to-wake transitions were observed for analysis, but this is inconsistent with mouse sleep physiology.

After eliminating recordings without interpretable EMG recordings and manually editing REM and wake classification, we identify many more REM-to-wake transitions. We now display jRGECO and GRAB-Ado dynamics around REM-to-wake in Figure 6I and 7G.

b. Second, we know from previous work in other cortical areas—and the authors state—that astrocyte intracellular calcium activity is highest during wakefulness and lower during REM sleep. In addition, the calcium activity during REM sleep is similar to NREM sleep, if not lower. However, the data in this manuscript are inconsistent with this previous work, which the authors acknowledge.

Reviewer 1 also made this observation. Please see our response to Reviewer 1's comment 5.

c. Third, previous work shows wake onset immediately after REM sleep is associated with large increases in astrocyte calcium activity that generally surpass that seen in NREM-to-wake transitions and sustained wakefulness. The data shown in Figure 5B depict the largest increases in astrocyte calcium are associated with REM sleep episodes/transitions. Based on previous work, I strongly suspect that these large calcium events instead occur during wakefulness rather than REM or NREM sleep as reported in this manuscript.

After reanalyzing our *in vivo* data, we agree that our submission 1 calcium results were likely skewed due to contamination of REM classification with wake bouts. We now only see a small increase in calcium at REM onset shown in Figure 6H (previously Fig 5) and we see a robust increase in calcium at REM-to-wake transitions (Fig 6I). Additionally, we no longer find any differences in WT and cKO calcium activity around REM transitions. Reviewer 2 also points out that previous studies have shown larger calcium increases at REM-to-wake than at NREM-to-wake. However, we find that the calcium increase at REM-to-wake is not as large as that observed at NREM-to-wake transitions. This could reflect intra-cortical astrocyte heterogeneity[5], which would also be consistent with the observation that REM-specific neuronal activity is higher in relative to other somatosensory cortices[3].

d. Related to the above point, I suspect the peaks shown in Figures 5G and 5H also likely reflect REM-to-wake transitions. For 5G, a peak occurs around 30 seconds after the denoted REM sleep onset. REM sleep episodes in mice last approximately 60 seconds on average. However, shorter REM episodes (e.g., 10 seconds and longer) are also common. It is not clear if the data shown in 5G and 5H only include REM episodes that are ≥ 60 s. But if shorter REM episodes are included, then these data likely reflect transitions to wakefulness (and perhaps subsequently to NREM sleep). REM-to-wake-to-NREM transitions are seen within a 60-s window in mice, and the pattern of astrocyte calcium activity in 5G

seems like it could reflect such a transition pattern. I also suspect the peak just following the denoted NREM onset in 5H also reflects activity during wakefulness.

Based on our reanalysis, we agree that NREM-to-REM and REM-to-NREM transition data in submission 1 was likely contaminated with wake periods, leading to the patterns that Reviewer 2 points out above. We now see a clear calcium increase at REM-to-wake transitions that peaks around 20s post wake-onset and observe very little calcium activity during REM. That said, we still see slightly elevated calcium at REM-onset in both genotypes (Fig 6H). We believe it is unlikely that wake contamination is driving this effect since EMG was used for all REM classification.

To avoid cross-contamination across state transitions, we implemented strict duration criteria: only wake, NREM, and REM bouts that met minimum length requirements were included in the transition analyses. Specifically, NREM/wake transitions spanned 400s, NREM-to-REM spanned 120s, and REM-to-wake spanned 60s, as now stated clearly in the figure legends for Figures 6 and 7.

e. If EMG was not used to help distinguish between wakefulness and NREM, I also wonder if the ~30-s decrease in astrocyte calcium after NREM onset denoted in 5F might be a period of quiet wakefulness.

We believe this is unlikely after re-scoring sleep/wake.

f. Finally, the authors state that REM sleep was sometimes determined without EMG but instead used the criteria that the bout had to follow a NREM bout and had to be ≤ 200 seconds. However, these criteria are equally applicable to wakefulness in mice. Therefore, this approach is prone to error.

This is an important critique, which we have thoroughly addressed by manually classifying REM periods based on the methods described in the introductory section of this document.

Given the inconsistencies between previous work and the current data, please compare the autoscoring against visual/manual scoring by an experienced experimenter to 1) validate (what seems to be) a relatively new approach for this team with respect to sleep analysis and 2) verify the accuracy of the currently reported state scorings. Without these data, it is difficult to accurately interpret the data presented in this manuscript.

Authors Maxine Levesque and Drew Willoughby made major contributions to the HMM and manual REM classification criteria used in our resubmission. Having worked in a human sleep laboratory, Maxine has extensive knowledge around best practices for EEG and EMG signal processing and thus made critical contributions for designing the HMM scoring pipeline. Drew Willoughby helped develop our REM reclassification criteria based on discussions with members of the Ganguly lab at UCSF. The Ganguly lab has carefully considered how to best score mouse sleep behavior – evident in their sleep-focused publications[12–15]. Thus, we believe that we had sufficient expertise driving the design for the HMM automated classifier and manual REM reclassification pipeline. Please see Response Figures in the introductory section (pages 1–2) for verification of our state scorings.

2) Related to #1 and the multitaper spectral analysis, I wonder if a smaller window size would provide a more accurate starting point for autoscoring. From my understanding, multitaper spectral analysis has been used primarily on human EEG data. Because human EEG is more stable (i.e., fewer state transitions), we typically scored human EEG in 30-s epochs. This stability is in contrast to mice which have more frequent state transitions and are therefore scored using 4-s or 10-s epochs. Therefore, a window size of 4 s or 10 s might be more appropriate for this work to improve temporal resolution especially given that the EEG-determined states are being compared against continuous changes in calcium and adenosine.

We appreciate Reviewer 2's thoughtful suggestion regarding epoch length in multitaper spectral analysis. Indeed, shorter epochs (e.g., 4–10 s) can enhance temporal resolution for mouse EEG, which typically exhibits more frequent state transitions compared to human EEG. However, window length has a tradeoff with frequency resolution and because it was critical for our analysis to specifically resolve delta-band activity between 0.5 and 4 Hz, we chose a window length of 30 s, giving us a frequency resolution of 1 / 30 = 0.0333 Hz. We mitigated the impacts this might have on segmentation by using a window slide of 1 s, allowing transitions to be identified with finer granularity than individual 30 s epochs. While we acknowledge the importance of the reviewer's note, that the window size of 30 s does introduce blurring between each 1 s window slide which reduces the effective time resolution of state segmentation, we determined that this choice was appropriate here because of the critical need for spectrally isolating delta-band activity, which was used to score NREM and wake periods via the HMM. The seconds-level temporal resolution should be sufficient for analysis of astrocytic jRGECO and extracellular GRAB-Ado, which are known to fluctuate on a tens of seconds timescale[5,16,17].

3) It is unclear how long the duration of EEG/EMG recordings were for each experiment described in 75, Figure 6, and Figure 7. The methods state 195 min recordings were captured under Fiber photometry and electrophysiology recordings. Was this true for experiments in Figure 6? Also, it seems that Figure 5 shows examples of ~250 min recordings.

We have clarified recording lengths in the Methods:

"*Experimental recordings lasted >195 min (final recording lengths were slightly shorter after signal processing and noise clipping) and each animal underwent recordings at least 1–2 times per week until 5-weeks post-surgery when animals were euthanized and perfused for immunohistochemistry to validate implant locations and viral expression.*"

After signal processing Figure 8 recordings were on average 192 ± 7 (std) minutes with 2 outliers: 171 and 215 minutes. Figure 6 and 7 recordings were on average 190 ± 9 (std) minutes and had 2 outliers: 160 and 250 minutes. These shorter recordings likely exhibited noise or movement artifacts that were clipped towards the beginning of the recording. Bout frequency was normalized to recording length to account for these outliers and slight variations in recording length.

4) Further detail regarding the process for transferring mice to recording cages and connecting mice to cables will be helpful. More specifically, how long were mice allowed to habituate to the recording environment and tether before each recording session? The Methods state mice habituated for at least one 2-h session before recordings began, but was this habituation period given before every recording?

We have added these details to the Methods section, which now reads:

"Animals were given 2 weeks post-surgery for recovery and viral expression. They were then habituated in a freely moving recording set up for at least one 2-hour session on the day or two before experimental recordings began. The recording chamber was a plexiglass cylinder (height = 8 in; diameter = 10 in) filled with bedding from the animal's home cage, which was cleaned with 70% ethanol between all habituation and recording sessions. For each experimental recording, the chamber contained fresh bedding material and the mouse's nest, which was transferred from its home cage. 1-2 recordings were done per day with the first starting ~7 ZT and the second ~10 ZT (mean recording time of day rounded to the nearest 0.5 hour was 9 ZT with a range of 6.5-11.5 ZT). On recording days, mice (Fig 6–8 and S4, S6–7) were plugged in and recordings were started immediately."

If not (and even if so), is it possible that H1R cKO mice need more time to habituate for Figure 6 experiments? If cKO mice needed longer to settle into the recording space, this could potentially account

for the increased time spent in wakefulness. Therefore, increased time in wakefulness might be related to anxiety, for example, rather than sleep/wake regulation. Hypnograms shown in Figures 5B and 7B show that cKO mice spend more time in wakefulness at the beginning of the recording compared to WT. If the data are available, calculating onset to NREM sleep after being connected to the EEG/EMG tether/preamplifier could help answer that question.

It is true that increased time in wakefulness might reflect differences in time before first sleep bout, which could be explained by differences in anxiety. Recordings were started immediately after plugging in a mouse's head mount, but we did not record the time between placing the animal in the recording chamber and starting the actual recording, making it difficult to assess time before first sleep bout. That said, we appreciate that alternate explanations for the sleep/wake phenotype should be addressed, so we have added this to the Discussion:

"Alternatively, considering H1R is linked to anxiety regulation (54,99,100), increased wakefulness and reduced REM sleep could reflect increased anxiety in cKO mice rather than changes to sleep/wake architecture regulation."

5) The authors state that all experiments occurred during the light period in the Methods. I have a few concerns regarding the time-of-day during which the experiments were performed:

a. First, this is an important detail that should be mentioned in the Abstract, Results, and Discussion sections.

We have added the circadian phase and time of day in ZT throughout the manuscript (Results and Methods). While we have not added it to the Discussion, we clarify the influence of recording time on sleep/wake phenotypes in the Results section.

b. The authors should state more precisely when during the light period (e.g., Zeitgeber time) these experiments were performed. Time spent in states, distribution of states, EEG spectral power, and astrocyte calcium activity differ across the light period, so this can impact interpretation of the data if time-of-day is not carefully considered. If experiments were spread across the light period, was this timing at least balanced across conditions for each experiment?

Yes, timing was relatively balanced across conditions for all sleep/wake recordings. Mean recording time of day (rounded to nearest 0.5 hours in ZT) for the WT and cKO data in Figure 6 was 8.5 ZT and 8.0 ZT, respectively. For all WT and cKO data, mean time was 8.5 ZT and 9.0 ZT, respectively. For all recordings, the time of day ranged from 6.5 ZT to 11.5 ZT. The following table shows how recording time of day was relatively balanced across experimental cohorts:

Experiment	Before 9.0 ZT	After 9.0 ZT
WT cohort in Figure 6	65% recordings	35% recordings
cKO cohort in Figure 6	75% recordings	25% recordings
WT cohort in Figure 5, S5, S7	55% recordings	45% recordings
cKO cohort in Figure 5, S5, S7	67% recordings	33% recordings

We have added the following to the Methods:

"1-2 recordings were done per day with the first starting ~7 Zeitgeber time (ZT) and the second ~10 ZT (mean recording time of day rounded to the nearest 0.5 hour was 9 ZT with a range of 6.5-11.5 ZT)."

Additionally, we have explicitly discussed how time of day might influence sleep/wake phenotypes in the Results:

"We also note that recording start times are similarly represented across WT and cKO groups in both the ipsilateral and contralateral cohorts, with 60% of all WT recordings and 71% of all cKO recordings starting before 9.0 ZT and the remaining recordings starting after 9.0 ZT. Since a larger fraction of cKO recordings start before 9.0 ZT, close to the middle of the light period or the inactive circadian phase, we might expect to see more sleep in the cKO cohorts. However, the cKO mice exhibit more wakefulness. This observation strengthens the overall conclusion that astrocyte-specific H1R deletion in the cortex has a wake-promoting effect."

6) Besides what's mentioned in #4 & 5, I wonder if time-of-day could account for the differences in time-in-state between Figure S5 and Figure 6. The fact that all mice are spending more time in wakefulness compared to NREM sleep in Figure 6B makes me wonder if these recordings started near light onset or at the end of the light period. This can be clarified by addressing #5. Or perhaps there was a disturbance in the room that prevented sleep.

I also think that time-of-day and/or behavioral changes unrelated to sleep/wake regulation are more likely to account for these differences than EEG electrode placement. The authors speculate that placing the EEG electrode ipsilateral (rather than contralateral) to the AAV injection accounts for the differences with Figure S5 and reflects local sleep. However, a change in EEG placement is unlikely to result in a change in overall behavior. Placing EEG electrodes over different parts of the cortex can change the spectral qualities of the EEG, but I am skeptical that we'd see an EEG electrode over one part of the cortex show > 20% difference in vigilance state from an electrode over another part of the cortex in mice when EMG can be used to help confirm behavior/state. Therefore, EMG should be used to more accurately define sleep/wake states.

If there are EEG spectral differences that do not align with other behavioral measures like EMG, that is very interesting! But it would be best to discuss this state characterization in a more nuanced way than how we typically describe sleep/wake behavior as you'll have something more akin to a dissociated state.

We appreciate Reviewer 2's point that electrode placement is unlikely to account for differences in sleep/wake behavior. Analysis of re-scored sleep/wake data still reveals distinct sleep/wake dynamics in the ipsilateral- and contralateral-EEG cohorts. While we no longer find a significant genotype-specific difference in NREM sleep time for contralateral cKO mice (Figure S7B), we still see a difference in % time asleep when comparing ipsilateral and contralateral cohorts. Figure S7B shows that contralateral WT and cKO mice spend 53 and 56% time in NREM, respectively, while Figure 8B shows that ipsilateral WT and cKO mice respectively spend 37 and 31% time in NREM. We also find that the contralateral cohorts spend more time in REM sleep relative to the ipsilateral cohorts.

While recording time of day can affect sleep/wake behavior, e.g. mice are expected to sleep more during their inactive (light) phase, time of day does not appear to explain the differences in ipsi- and contralateral cKO sleep/wake phenotypes. This is illustrated in figure below, which plots the percentage of time spent in wake, NREM, and REM sleep against the recording start time (rounded to nearest 0.5 hour in ZT). For instance, in contralateral cKO mice, NREM sleep consistently accounts for around 50% of the recording, regardless of start time, whereas in ipsilateral cKO mice, NREM percentages cluster between 30–40%.

In submission 1, we suggested that relative EEG screw placement could account for the different sleep/wake dynamics in contralateral and ipsilateral cohorts. After considering reviewer comments, we believe a likely explanation for different sleep/wake phenotypes, and specifically the relative sleepiness of the contralateral mice, is the additional photometry fiber cable tethered to their head mounts. It is possible that this extra cable adds physical strain that decreases activity and promotes stillness, which could

increase likelihood of sleep these mice. We have added this possible explanation to the manuscript (see second to last paragraph of Results section titled *Astrocytic H1R deletion in cortex shapes wake and REM sleep dynamics*).

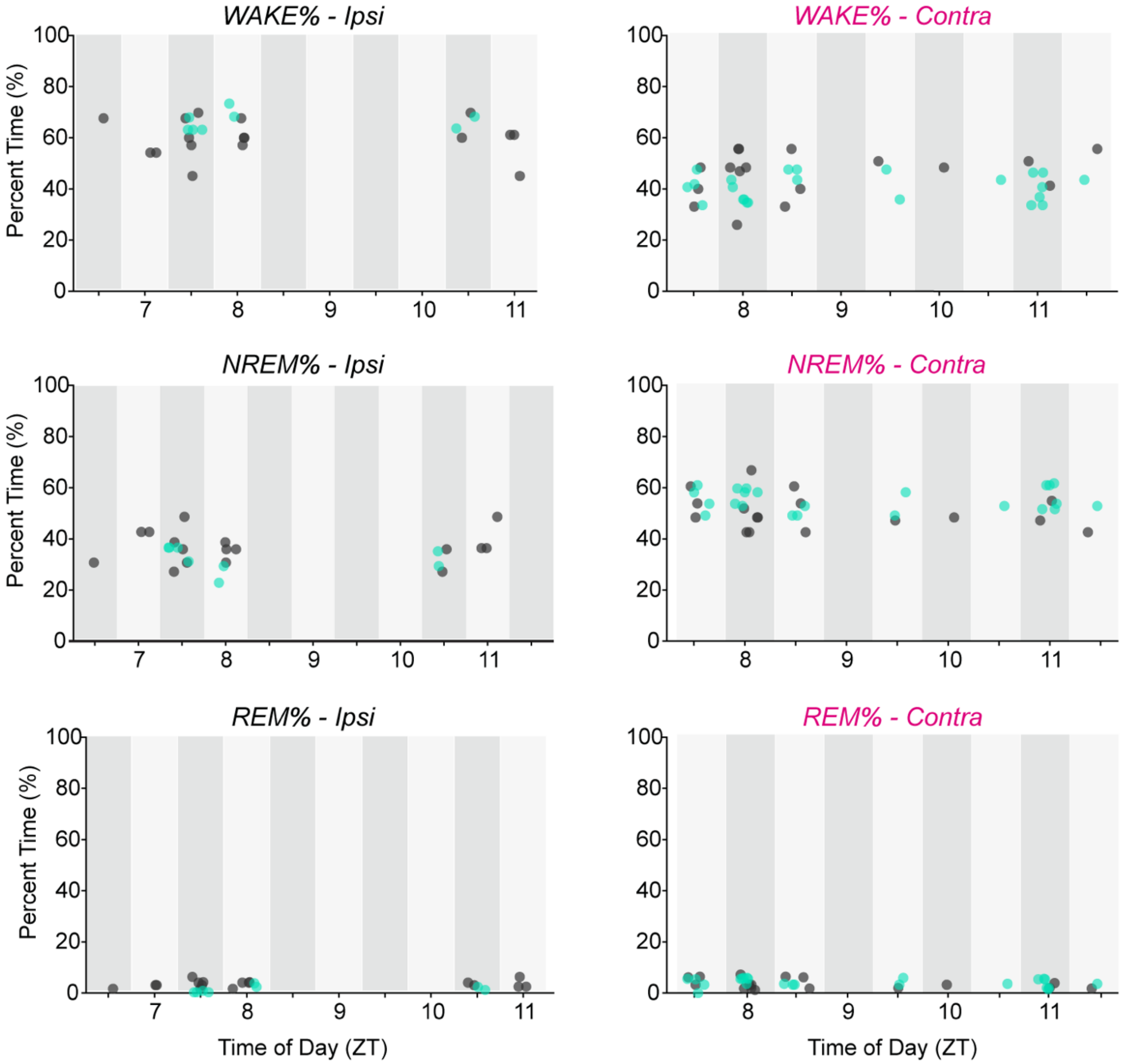

7) What was the rationale for conducting these studies during the light period? Because histamine is wake-promoting and we'd expect knocking out H1R to decrease wakefulness, it seems like this prediction would be best tested during the dark period when wake propensity is higher in mice. The increased astrocyte calcium activity during light-period wakefulness is interesting, but it might not generalize to the dark period since we often see manipulation affect sleep/wake parameters in one phase but not the other. It might be helpful to mention this in the Discussion.

We conducted our study during the light period for the following reasons:

1) To be consistent with other cortical astrocyte-focused sleep/wake studies done in the light period.[18–20]

2) To ensure we captured sufficient time in sleep for analysis, since we were using a freely moving set up and anticipated a lot of movement during the animals' active (dark) phase.

While we believe in this rationale, we also agree with Reviewer 2's point. We now present the REM calcium result in the context of recording time of day, as stated in the Discussion: "*both the WT and cKO animals exhibited a small increase in astrocyte Ca^{2+} at NREM-to-REM transitions when measured during the circadian light phase*".

MINOR

8) The authors speculate that calcium activity in cKO cells after histamine application might be due to being connected to the syncytium or neuronal H1R-driven responses. But is it also possible that histamine is activating other histamine receptors even if H1R is enriched on astrocytes?

We think this is unlikely since application of an H1R antagonist blocks Ca^{2+} responses (Figure 2C), which suggests that other astrocyte expressed HA receptors do not contribute to HA-triggered Ca^{2+}.

9) Figure 4B: The text indicates that norepinephrine was added to slices after histamine application and that these applications were separated by at least 10 min (pg. 6, lines 33-34; pg. 7, lines 6-7, pg. 11, line 20). However, Figure 4B seems to indicate histamine was applied continuously and norepinephrine was added later, but during, histamine application.

Once added, HA was indeed circulating in the ACSF bath for the remainder of the experiment. NE was added >10 minutes after this initial HA addition. We have edited the Results text to make this clear: "*NE was added to the recirculating ACSF bath least 10 minutes after the initial HA addition*".

10) Please state how many slices from how many animals were used in Figure 4 and S4 experiments.

Data in Fig 4B, D–E and S4C–E were collected from 9 slices and 3 animals (3 slices per animal). Data in Fig 4C, F were collected from 5 slices and 2 animals. We have added this to each figure legend.

11) Please state how many animals were used in Figure 5 experiments.

Data shown in Figure 6 (previously Figure 5) includes 7 WT mice and 9 cKO mice. We have added this information to Figure 6A.

12) How were sleep/wake bouts defined? Was a minimum duration threshold used (e.g., at least 30 s for wakefulness)? Please define this in the methods.

We have updated all sleep/wake scoring criteria in the manuscript. The Methods now read:

"For sleep scoring, multitaper spectral analysis (number of tapers = 29; window = 30 s; step size = 1 s) was performed on EEG data (102) to determine average delta (0.5–4 Hz) power over time. These data were used to train a Hidden Markov Model (hmmlearn, GitHub) to infer NREM and wake states in each recording. For data in Figure 8, EMG gamma power (65-100 Hz) was also used to infer NREM and wake bouts. For animals with photometry (Fig 6–7, S4, S6–7), EMG data was excluded from the HMM in due to noise artifacts in photometry recordings, which could be interpreted by a human experimenter but not by the automated model. In the HMM model, wake and NREM periods were scored with 1 and 0, respectively. To identify behavioral states with high confidence, 100 iterations of the HMM were averaged. NREM and wake bouts were then identified based on HMM scores across the EEG recording (NREM < 0.45; wake > 0.55) and minimum bout durations (NREM ≥ 100s and wake ≥ 30s). HMM-

predicted states were then plotted alongside z-scored theta/delta ratio and z-scored EMG gamma power to facilitate manual REM bout detection. Then, HMM detected wake bouts that met the following criteria were manually reclassified as REM if they (1) followed NREM period, (2) exhibited a theta/delta peak > median + 2 standard deviations, and (3) showed minimal to no EMG gamma power. REM bouts—defined by a theta/delta peak with minimal EMG gamma—were often followed by a brief increase in EMG gamma power, consistent with brief awakenings after REM sleep in mice. These were manually scored as wake. Using this method, 91–96% (Fig 8 and S7) of each recording was classified as wake, NREM, or REM, leaving 4–9% unclassified."

13) Pg. 8, lines 13-14: I wonder if the differences between in vivo and ex vivo calcium activity data might, at least in part, be due to different measurements used. In vivo data are described in terms of peak amplitude and peak frequency, but ex vivo data are described in terms of active pixels.

This is a good point. A more analogous analysis method to the *in vivo* jRGECO peak amplitude and change in jRGECO fluorescence at state transitions is quantification of ΔF/F in individual astrocytes in cortical slices. We have added this *ex vivo* analysis in the new main Figure 5 and highlighted the parallels between *ex vivo* results shown in this figure and the *in vivo* results shown in Figure 6.

14) Pg. 8, line 11: I think 5F is supposed to be 5G.

15) Pg. 8, line 24: I think 5E is supposed to be 5F.

16) Pg. 8, line 25: I think Fig. 5E, G is supposed to be Fig. 5E, F.

17) Pg. 8, line 26: I think 5F is supposed to be 5G, H.

We thank Reviewer 2 for their attention to detail and these specific manuscript text edits (points 14-17). We have double checked figure labels and mentions throughout the main text of our manuscript.

18) Figure S6C: should the green label be something different from Cre-GFP? Also, S6D seems to show Cre expression in the contralateral cortex.

Yes, this is a typo. It should read "GFP" only. We have corrected this in Fig S5C, E–F (previously Fig S6). We do see some contralateral Cre-GFP expression in the mouse brain imaged in Fig S5D, which can be more clearly seen in a different brain section shown below. This expression is minimal but partly informs our decision to remove the claim that uni-hemispheric astrocytic H1R deletion can drive changes in contralateral sleep/wake dynamics.

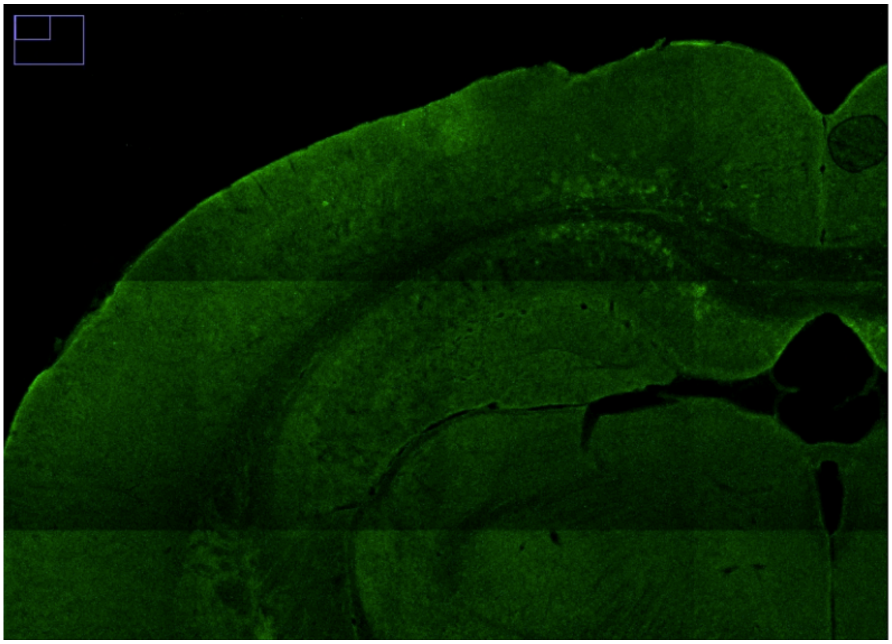

19) When no Cre virus was injected (e.g., Figure 5), was anything added to supplement the volume so that WT and cKO were injected with the same volume? If not, please edit the Methods to reflect this detail.

When no Cre virus was injected, animals were still injected with a viral mixture (for example, viruses for GRAB-Ado and jRGECO). All animals were injected with a total volume of 1000 nl, as stated in the Methods:

"*a craniotomy window was opened over left V1 and we subsequently injected -0.2 mm below cortex with 1000 nl viral vector mixtures at 2 nl/sec*".

We clarify viral mixture ratios for each experiment in the following sentences of this Methods section.

20) Pg. 31, line 17: Is a ~5 mm diameter tip correct?

This is not correct – it is a typo. It should read 5 microns (μm). We have corrected this in the Methods.

21) In the Methods, please state the vendor and product numbers for all of the AAVs. If they are custom, please state that.

We have added these details to the Methods.

22) Pg. 35, lines 33-34: As stated, it reads like mice were anesthetized with buprenorphine and carprofen.

We have edited this in the Methods. It now reads "*mice were anesthetized with vaporized isoflurane (1–1.5% vol.) and administered 0.05 mg/kg buprenorphine (s.c.) and 5 mg/kg carprofen (i.p.)".*

23) Pg. 36: Please state the AAV titers used for the in vivo experiments. If they are the same as ex vivo experiments, please state that.

In the Methods, we have added missing AAV titers and clarified when the same virus was used in *ex vivo* and *in vivo* experiments.

Reviewer #3

This is an interesting study. Charlotte R. Taylor claimed that histamine increases astrocyte intracellular Ca^{2+} concentration via H1R in V1. Although V1 astrocyte-specific H1R deletion minimally affects sleep/wake architecture or cortical oscillations, they revealed a significant decrease in extracellular adenosine levels during REM sleep rather than wakefulness. However, there are major issues that need to be corrected before appropriate interpretation of the results can be done.

Major:
1) Previous studies suggest that histamine neural activity is high during wakefulness and low during sleep. However, is the actual extracellular histamine concentration in V1 also high during wakefulness and low during sleep? This is the key question of this study, so why not measure it using a GRAB sensor?

We appreciate Reviewer 3's thoughtful suggestion. We have addressed this concern by recording fiber photometry GRAB-histamine (GRAB-HA) signal in V1 across sleep/wake. In new Figure S4, we present analysis of 6 GRAB-HA fiber photometry recordings that had interpretable EMGs recordings, which shows that extracellular HA in V1 is high during wake and low during sleep. Our results are consistent with the 2023 publication that characterized the GRAB-HA sensor demonstrating that extracellular histamine levels in prefrontal cortex increase with wake onset, decrease at NREM, and decrease further at REM.

Likely due to EMG signal processing methods (see paragraph 1, page 2 for more in-depth explanation), we were able to identify only 3 REM-to-wake transitions, which is insufficient for meaningful GRAB-HA transition analysis. We therefore only show transition dynamics for NREM-to-wake, wake-to-NREM, and NREM-to-REM. Nevertheless, our results are consistent with previously published cortical GRAB-HA dynamics, supporting the conclusion that V1-specific extracellular HA dynamics are high during wake, low during NREM, and lowest during REM.

2) In the astrocyte H1R cKO experiments, I find it difficult to understand why the effects on sleep/wake alternation differ between Figure 5 and Figure 6. The authors concluded that "Thus, uni-hemispheric H1R deletion in cortical astrocytes could cause differences in time awake between hemispheres, consistent with local sleep differences observed across cortex," citing the study by Vyazovskiy, V. V. et al., as supporting evidence. However, their findings refer to differences on the scale of hundreds of milliseconds, which may influence cortical oscillations but not the sleep/wake state itself. Moreover, the cited study was conducted on rats, not mice, making its applicability less convincing.

Please see our response to Reviewer 2's comment 6.

3) The authors assessed the specificity of H1R cKO in astrocytes using immunohistochemistry. However, there is no information on the number of V1 astrocytes that undergo H1R deletion. Additionally, there are no reports on the variability in the H1R deletion among different animals. This is relevant to major comment #2), as I am concerned that the extent of H1R deletion may also influence sleep/wake effects in individual animals. Correlating the extent of H1R deletion with the sleep/wake outcomes would be valuable. Without this information, it is difficult to accurately evaluate the role of H1R in V1 astrocytes in sleep/wake regulation.

We agree that correlating sleep/wake behaviors with the extent of H1R deletion in individual animals could provide additional insight into the role of cortical astrocytic H1R signaling in sleep/wake regulation. While we did not perform direct mRNA quantification of H1R deletion in the *in vivo* animals, we performed a coarse analysis of Cre or GFP (sham virus) expression levels (zero, low, high) in the ipsilateral cohort (animals included in Figure 8), as illustrated in the figure below. This analysis includes three additional mice that received virus injections but exhibited no detectable transduction. These zero-

expression animals were excluded from the primary manuscript analyses but are included here to provide context.

In the figure below, percent time spent in each vigilance state is plotted by genotype and estimated virus expression level. Notably, the Cre-injected mice with zero expression cluster with WT controls, consistent with a lack of H1R deletion. However, we do not observe a clear separation in sleep/wake metrics between cKO mice with low versus high levels of Cre expression. This suggests that the sleep/wake phenotype in H1R cKO mice is not strongly dependent on the extent of Cre expression, at least within the range of Cre expression levels captured here.

Given this lack of a dose-dependence, we did not pursue further quantification of H1R deletion.

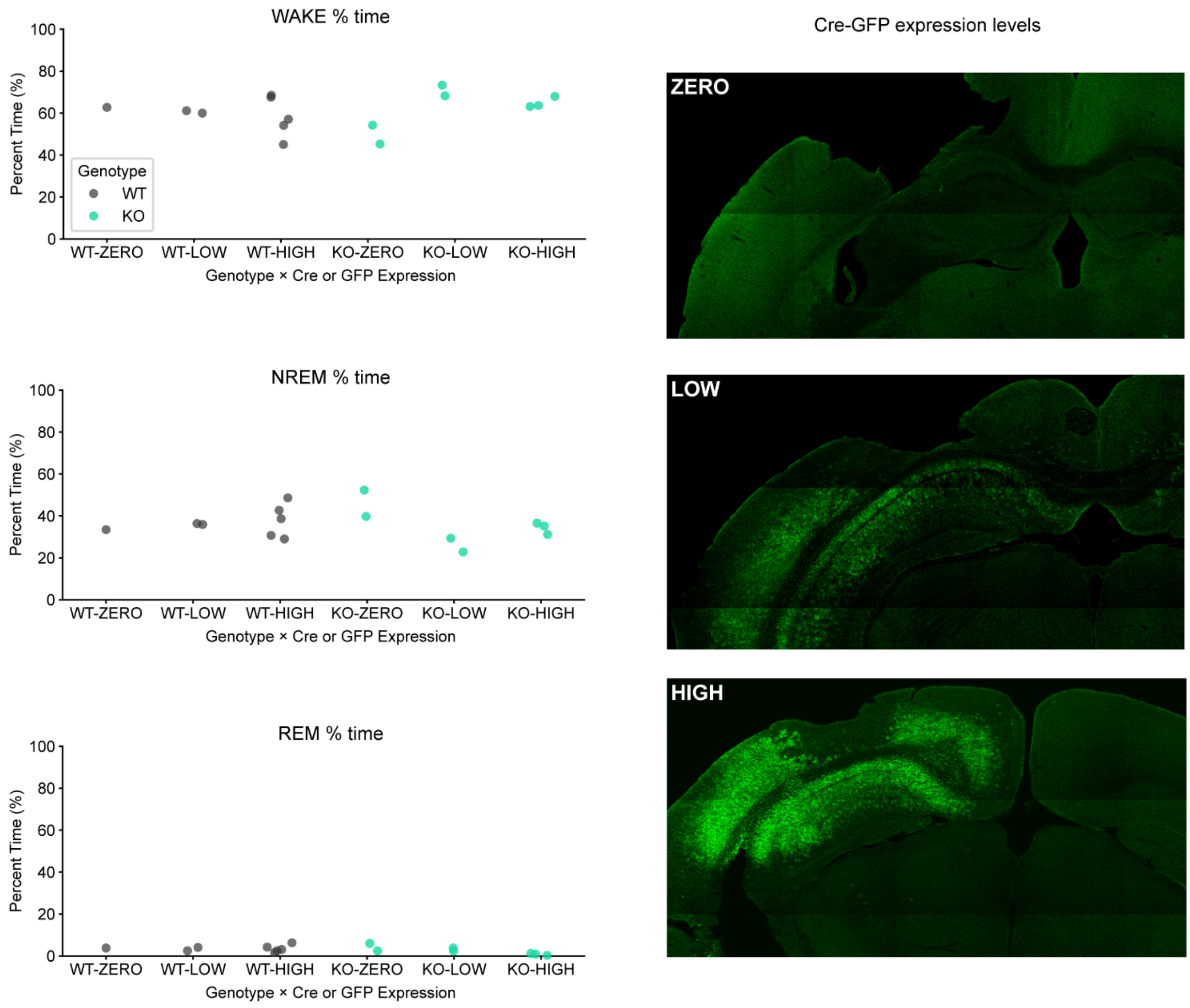

4) On page 7, the authors claimed that "However, neighboring WT astrocytes exhibited attenuated responses to NE after HA stimulation (Fig. 4B, D). While this may be due to exhaustion of Ca2+ stores since we saw no difference in WT and cKO Ca2+ responses to NE when slices were not previously stimulated with HA (Fig. 4C, F; Video 4), we think it is unlikely because NE responses in cerebellar astrocytes recover within 4 minutes after a single preceding NE stimulation15, and HA and NE addition were separated by at least 10 minutes here. Instead, our results could reflect HA-triggered changes in astrocyte activity that outlast acute H1R activation, consistent with the observation that the decrease in NE-triggered WT responses relative to neighboring cKO responses is not correlated with the time

between HA and NE addition (Fig. 4E). Together, these data show that HA directly activates astrocytes via H1R, leading to acute Ca2+ elevations in H1R-expressing astrocytes and potential regulation of subsequent neuromodulator responses." To support this assumption, it is necessary to conduct an experiment of repetitive NE application on V1 astrocytes.

We appreciate Reviewer 3's request for an additional experiment to test our claim that HA stimulation of H1R regulates astrocyte responses to other neuromodulators; we are very interested in this finding! We decided to test the effect of minimal H1R stimulation on NE response magnitudes. We quantify response magnitudes by comparing ΔF/F in WT and cKO astrocytes. We applied this same analysis method to the experiment shown in Figure 4C and F. This revealed that H1R shapes astrocyte responses to NE even before HA application, which establishes that genotype-specific differences in NE responses are not simply due to Ca^{2+} store exhaustion. We are excited about these results and now present them in new main Figure 5. We thank Reviewer 3 for this additional experiment request as we believe that this experiment has strengthened our claims and, in particular, revealed interesting parallels between our *ex vivo* and *in vivo* Ca^{2+} results which is discussed in the manuscript.

Minor:

1) In supplementary figure 1A, why is the increase in Ca2+ concentration occurring before time 0?

This is due to how the data is binned (at 10s in Fig S1A). Figure below shows traces when using 1s bins (instead of 10s).

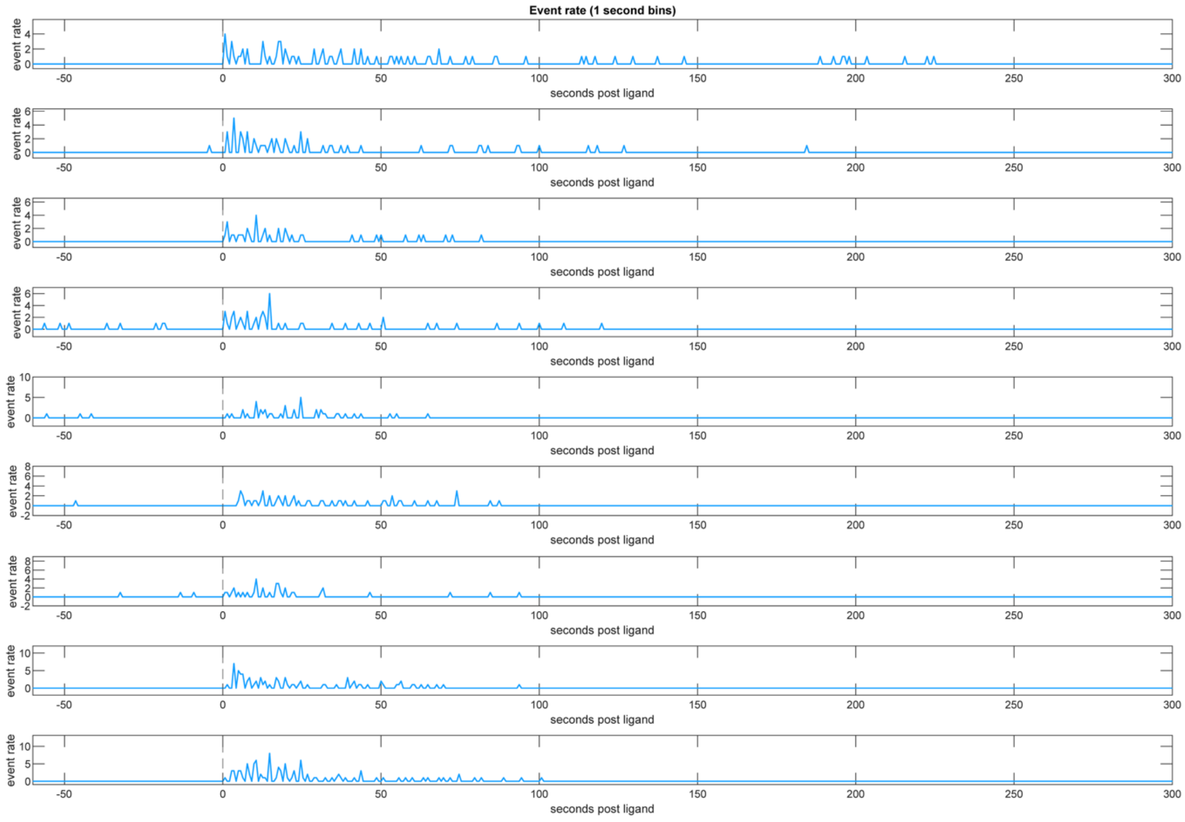

2) Are all astrocytes expressing H1R? Or is it differentially expressed in different brain regions? Please add this information.

Brain-wide and astrocyte-specific H1R mRNA has been quantified by Allen Brain Institute. In their analysis, astrocytes throughout the brain express H1R at different levels. Cortical astrocytes exhibit mid-level H1R expression. Hippocampal astrocytes express comparatively low levels, while thalamic and midbrain astrocytes express high levels of H1R (see screenshot from Allen Brain Cell Atlas below). We now cite this work on pages 3–5 in our manuscript.

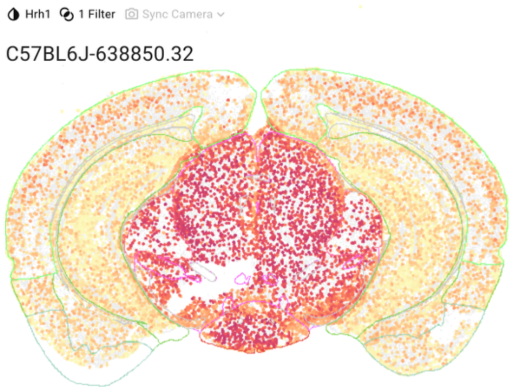

3) In Figure 5, isn't the REM offset simply due to the lower increase in Ca2+ during REM compared to WT? This could explain why the authors do not detect any change in the Wake-to-NREM transition. If the authors are unable to present REM-to-Wake results, they should be more cautious in their interpretation of the NREM offset.

After manually rescoring REM sleep, we no longer see the genotype-specific differences in Ca^{2+} dynamics around REM transitions that Reviewer 3 refers to here. We also identify far fewer REM-to-NREM transitions after rescoring and have not analyzed these transitions (as noted in introduction of this document). That said, we appreciate that our normalization method affects interpretation of our results. We therefore emphasize that we have quantified <u>changes</u> in Ca^{2+} (and GRAB-Ado) across transitions, because it is difficult to determine the absolute level of baseline Ca^{2+} from z-scored photometry measurements.

4) When did the authors record EEG/EMG to validate sleep/wake state and cortical oscillations in ZT? Please mention this in the results and the methods sections.

Please see our response to Reviewer 2's point 5.

5) In supplementary figure 6A, according to the AAV injection site, it appears to be V2 rather than V1.

Although we targeted stereotaxic virus injections to V1, we observed virus transduction spread beyond V1 into surrounding cortical areas. We added the following to the Results section to accurately reflect the extent of virus spread:

"*We found viral transduction localized below the EEG screw, with Cre or sham virus transduction evident across cortex and in hippocampus, but not in deeper subcortical regions that regulate sleep/wake dynamics (Fig S5C–H).*"

References

1. Weber, F. *et al.* Regulation of REM and Non-REM Sleep by Periaqueductal GABAergic Neurons. *Nat Commun* 9, (2018).
2. Sulaman, B. A., Wang, S., Tyan, J. & Eban-Rothschild, A. Neuro-orchestration of sleep and wakefulness. *Nat Neurosci* 26, 196–212 (2023).
3. Wang, Z. *et al.* REM sleep is associated with distinct global cortical dynamics and controlled by occipital cortex. *Nat Commun* 13, 1–17 (2022).
4. Simpson, E. H. *et al.* Lights, fiber, action! A primer on in vivo fiber photometry. *Neuron* 112, 718–739 (2024).
5. Pittolo, S. *et al.* Dopamine activates astrocytes in prefrontal cortex via α1-adrenergic receptors. *Cell Rep* 40, 111426 (2022).
6. Kjaerby, C. *et al.* Memory-enhancing properties of sleep depend on the oscillatory amplitude of norepinephrine. *Nat Neurosci* 25, 1059–1070 (2022).
7. Corkrum, M. *et al.* Dopamine-Evoked Synaptic Regulation in the Nucleus Accumbens Requires Astrocyte Activity. *Neuron* 105, 1036-1047.e5 (2020).
8. Zhang, W. T. *et al.* Spectral fiber photometry derives hemoglobin concentration changes for accurate measurement of fluorescent sensor activity. *Cell Reports Methods* 2, (2022).
9. Turner, K. L., Gheres, K. W., Proctor, E. A. & Drew, P. J. Neurovascular coupling and bilateral connectivity during nrem and rem sleep. *Elife* 9, 1 (2020).
10. Tsai, C. J. *et al.* Cerebral capillary blood flow upsurge during REM sleep is mediated by A2a receptors. *Cell Rep* 36, 109558 (2021).
11. Cahill, M. K. *et al.* Network-level encoding of local neurotransmitters in cortical astrocytes. *Nature* 629, 146–153 (2024).
12. Kim, J., Gulati, T. & Ganguly, K. Competing Roles of Slow Oscillations and Delta Waves in Memory Consolidation versus Forgetting. *Cell* 179, 514-526.e13 (2019).
13. Lemke, S. M. *et al.* Coupling between motor cortex and striatum increases during sleep over long-term skill learning. *Elife* 10, 1–25 (2021).
14. Kim, J. *et al.* Recovery of consolidation after sleep following stroke—interaction of slow waves, spindles, and GABA. *Cell Rep* 38, 110426 (2022).
15. Darevsky, D., Kim, J. & Ganguly, K. Coupling of Slow Oscillations in the Prefrontal and Motor Cortex Predicts Onset of Spindle Trains and Persistent Memory Reactivations. *The Journal of Neuroscience* 44, e0621242024 (2024).
16. Peng, W. *et al.* Regulation of sleep homeostasis mediator adenosine by basal forebrain glutamatergic neurons. *Science (1979)* 369, (2020).

17. Reitman, M. E. *et al.* Norepinephrine links astrocytic activity to regulation of cortical state. *Nat Neurosci* 26, 579–593 (2023).

18. Vaidyanathan, T. V, Collard, M., Yokoyama, S., Reitman, M. E. & Poskanzer, K. E. Cortical astrocytes independently regulate sleep depth and duration via separate GPCR pathways. *Elife* 10, 1–55 (2021).

19. Tsunematsu, T., Sakata, S., Sanagi, T., Tanaka, K. F. & Matsui, K. Region-Specific and State-Dependent Astrocyte Ca21 Dynamics during the Sleep-Wake Cycle in Mice. *Journal of Neuroscience* 41, 5440–5462 (2021).

20. Bojarskaite, L. *et al.* Astrocytic Ca2+ signaling is reduced during sleep and is involved in the regulation of slow wave sleep. *Nat Commun* 11, 3240 (2020).
